# Deciphering Symbiotic Interactions of “*Candidatus* Aenigmarchaeota” with Inferred Horizontal Gene Transfers and Co-occurrence Networks

**DOI:** 10.1128/mSystems.00606-21

**Published:** 2021-07-27

**Authors:** Yu-Xian Li, Yang-Zhi Rao, Yan-Ling Qi, Yan-Ni Qu, Ya-Ting Chen, Jian-Yu Jiao, Wen-Sheng Shu, Hongchen Jiang, Brian P. Hedlund, Zheng-Shuang Hua, Wen-Jun Li

**Affiliations:** a State Key Laboratory of Biocontrol, Guangdong Provincial Key Laboratory of Plant Resources and Southern Marine Science and Engineering Guangdong Laboratory (Zhuhai), School of Life Sciences, Sun Yat-Sen University, Guangzhou, People’s Republic of China; b School of Life Sciences, South China Normal University, Guangzhou, People’s Republic of China; c State Key Laboratory of Biogeology and Environmental Geology, China University of Geosciences, Wuhan, People’s Republic of China; d School of Life Sciences, University of Nevada Las Vegas, Las Vegas, Nevada, USA; e Nevada Institute of Personalized Medicine, University of Nevada Las Vegas, Las Vegas, Nevada, USA; f Department of Environmental Science and Engineering, University of Science and Technology of China, Hefei, People’s Republic of China; g State Key Laboratory of Desert and Oasis Ecology, Xinjiang Institute of Ecology and Geography, Chinese Academy of Sciences, Urumqi, People’s Republic of China; University of Pretoria

**Keywords:** “*Ca.* Aenigmarchaeota”, DPANN, symbiont, horizontal gene transfer, co-occurrence network, coevolution network

## Abstract

“*Candidatus* Aenigmarchaeota” (“*Ca.* Aenigmarchaeota”) represents one of the earliest proposed evolutionary branches within the *Diapherotrites*, *Parvarchaeota*, *Aenigmarchaeota*, *Nanoarchaeota*, and *Nanohaloarchaeota* (DPANN) superphylum. However, their ecological roles and potential host-symbiont interactions are still poorly understood. Here, eight metagenome-assembled genomes (MAGs) were reconstructed from hot spring ecosystems, and further in-depth comparative and evolutionary genomic analyses were conducted on these MAGs and other genomes downloaded from public databases. Although with limited metabolic capacities, we reported that “*Ca.* Aenigmarchaeota” in thermal environments harbor more genes related to carbohydrate metabolism than “*Ca.* Aenigmarchaeota” in nonthermal environments. Evolutionary analyses suggested that members from the *Thaumarchaeota*, *Aigarchaeota*, *Crenarchaeota*, and *Korarchaeota* (TACK) superphylum and *Euryarchaeota* contribute substantially to the niche expansion of “*Ca.* Aenigmarchaeota” via horizontal gene transfer (HGT), especially genes related to virus defense and stress responses. Based on co-occurrence network results and recent genetic exchanges among community members, we conjectured that “*Ca.* Aenigmarchaeota” may be symbionts associated with one MAG affiliated with the genus *Pyrobaculum*, though host specificity might be wide and variable across different “*Ca.* Aenigmarchaeota” organisms. This study provides significant insight into possible DPANN-host interactions and ecological roles of “*Ca.* Aenigmarchaeota.”

**IMPORTANCE** Recent advances in sequencing technology promoted the blowout discovery of super tiny microbes in the *Diapherotrites*, *Parvarchaeota*, *Aenigmarchaeota*, *Nanoarchaeota*, and *Nanohaloarchaeota* (DPANN) superphylum. However, the unculturable properties of the majority of microbes impeded our investigation of their behavior and symbiotic lifestyle in the corresponding community. By integrating horizontal gene transfer (HGT) detection and co-occurrence network analysis on “*Candidatus* Aenigmarchaeota” (“*Ca.* Aenigmarchaeota”), we made one of the first attempts to infer their putative interaction partners and further decipher the potential functional and genetic interactions between the symbionts. We revealed that HGTs contributed by members from the *Thaumarchaeota*, *Aigarchaeota*, *Crenarchaeota*, and *Korarchaeota* (TACK) superphylum and *Euryarchaeota* conferred “*Ca.* Aenigmarchaeota” with the ability to survive under different environmental stresses, such as virus infection, high temperature, and oxidative stress. This study demonstrates that the interaction partners might be inferable by applying informatics analyses on metagenomic sequencing data.

## INTRODUCTION

With advances in sequencing technologies and bioinformatic approaches, insight into the “unseen majority” prokaryotes has become possible, even when they inhabit complex microbial communities, leading to a tremendous expansion of known archaeal diversity (1–7). Among recently proposed major archaeal lineages, the *Diapherotrites*, *Parvarchaeota*, *Aenigmarchaeota*, *Nanoarchaeota*, and *Nanohaloarchaeota* (DPANN) superphylum has inspired considerable research attention, which has uncovered their surprisingly small genome sizes, lack of genes associated with core biosynthetic pathways (3, 8, 9), and extensive phylogenetic and functional diversity (10–12). “*Candidatus* Aenigmarchaeota” (“*Ca.* Aenigmarchaeota”), which represent the “A” of the DPANN superphylum, were first uncovered and named as the “Deep Sea Euryarchaeotic Group (DSEG)” (13). Later, based on single-amplified genomes (SAGs), this lineage was defined and proposed as a novel phylum (2). Other studies integrating metagenomic and metatranscriptomic sequencing revealed that this phylum lacks many essential metabolic pathways and may possess fermentative and symbiotic lifestyles (3, 8). However, our understanding of the metabolic characteristics, functional diversity, and potential host-symbiont interactions of “*Ca.* Aenigmarchaeota” is far from sufficient.

Here, we apply comparative and evolutionary genomics analyses on eight new metagenome-assembled genomes (MAGs) along with 15 publicly available genomes to fill these gaps. Our study reveals a symbiotic lifestyle for “*Ca.* Aenigmarchaeota” based on the absence of many genes involved in core metabolic pathways. Further analyses suggest that the occurrence of horizontal gene transfer (HGT) improves the competitiveness of “*Ca.* Aenigmarchaeota” by expanding their gene repertoires relevant to stress response and virus defense. We also integrate the HGT inference and co-occurrence network construction to reveal potential functional and genetic interactions between “*Ca.* Aenigmarchaeota” and other microbes.

## RESULTS AND DISCUSSION

### Phylogeny and distribution of “*Ca.* Aenigmarchaeota.”

**FIG 1:**
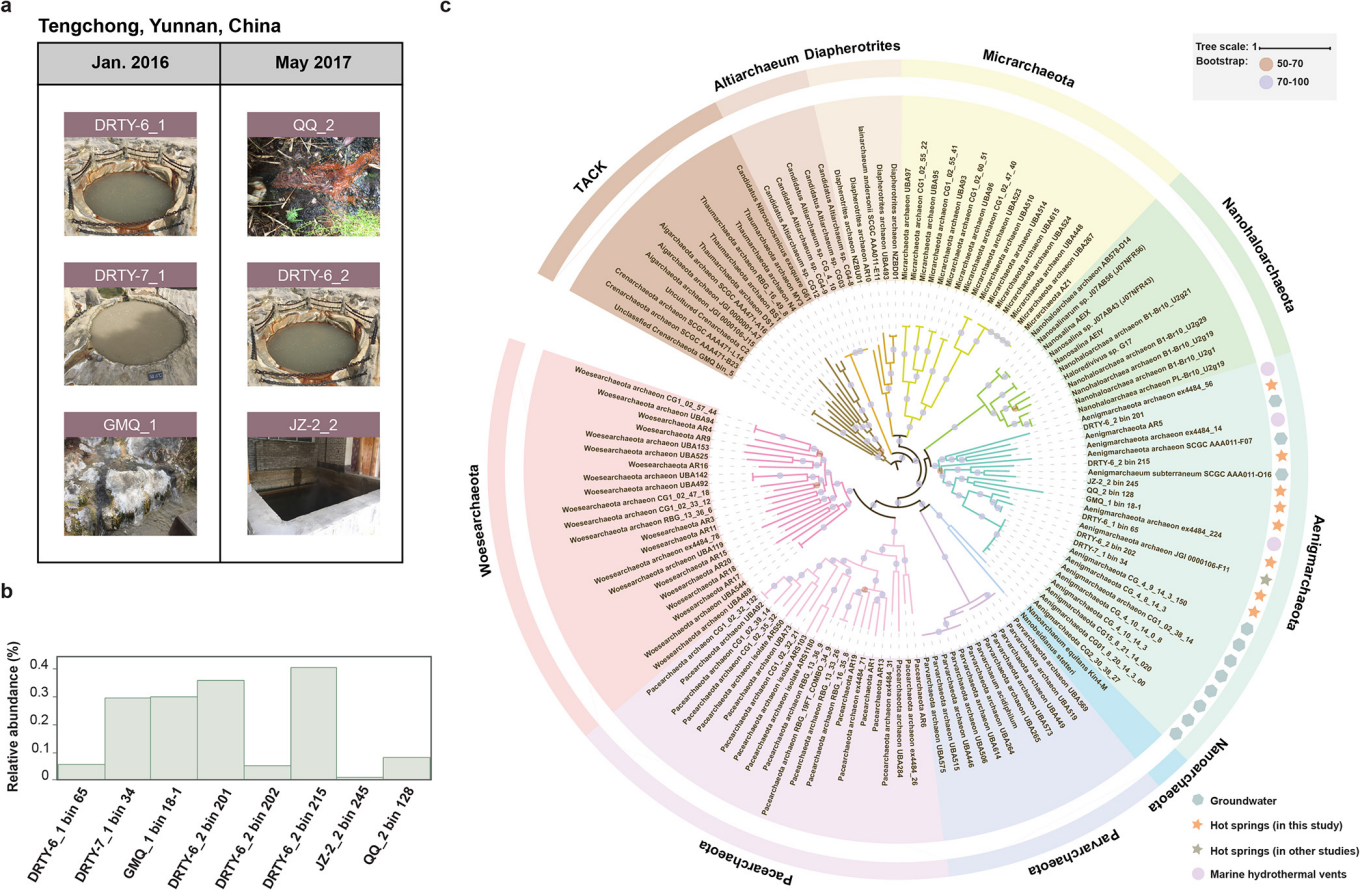
Phylogenetic analysis of reconstructed genomes of “*Ca.* Aenigmarchaeota” sampled from hot spring sediments in Yunnan, China. (a) Sampling sites of “*Ca.* Aenigmarchaeota” in Tengchong, Yunnan, China. Hot spring sediments from a total of five sites were collected in January 2016 and May 2017. (b) Relative abundances of “*Ca.* Aenigmarchaeota” MAGs were calculated by the coverage of the scaffolds of each MAG over the coverage of all the scaffolds of the corresponding metagenome. (c) Phylogenetic placement of the reconstructed MAGs. Maximum likelihood phylogeny of eight “*Ca.* Aenigmarchaeota” MAGs in this study and reference genomes from TACK and DPANN superphyla. Phylogeny was constructed based on a concatenated set of 16 ribosomal proteins with 1,000 bootstrap iterations.

Eight MAGs of “*Ca.* Aenigmarchaeota” were successfully reconstructed from five hot spring sediment samples collected in Tengchong county in Yunnan, China (Fig. 1a), including four from a single sample from Diretiyanqu-6 (DRTY-6) and one for each of the other springs Diretiyanqu-7 (DRTY-7), Gumingquan (GMQ), Qiaoquan (QQ), and Jinze (JZ-2) (Table 1) (14). “*Ca.* Aenigmarchaeota” represents a rare group in hot spring ecosystems with relative abundances of all MAGs of <0.4% (Fig. 1b). Most MAGs are of high quality, with completeness of >90%, nearly no contamination, and detectable 16S rRNAs and tRNAs (>21) (Table 1; Data Set S1 in the supplemental material) (15). Compared to MAGs of “*Ca.* Aenigmarchaeota” from other studies (Table S1), they have smaller genome sizes (0.64 versus 0.86 mega base pairs [Mbp]; Mann-Whitney *U* test, *P = *0.0003; Fig. S1) and a lower range of GC content (average 31.74% versus 38.59%; Mann-Whitney *U* test, *P = *0.012) (2, 3, 8); they also harbor a smaller number of genes (752 versus 1,070; Mann-Whitney *U* test, *P = *0.002), shorter average gene length (771 versus 683 bp; Mann-Whitney *U* test, *P = *0.0005), and remarkably high coding density (88 to 94.6%) and percentage of overlapping genes (∼20.6%) (Table 1). This is consistent with previous studies suggesting that thermophiles harbor small genome sizes as a result of genomic streamlining due to high fitness costs of life at high temperatures (16). Both whole-genome-based phylogenomic and 16S rRNA gene-based phylogenetic analyses revealed that the eight MAGs from this study branched within the phylum “*Ca.* Aenigmarchaeota” with high bootstrap confidences (Fig. 1c; Fig. S2). 16S rRNA sequences of “*Ca.* Aenigmarchaeota” were retrieved from the NCBI-nr database and used to illustrate the geographical distribution (see Materials and Methods). The results demonstrated that “*Ca.* Aenigmarchaeota” represented an evolutionarily diverse group that inhabits a broad range of ecosystems (Data Set S2), including freshwater (40.96%), marine water (27.71%), hot springs (7.63%), hydrothermal vents (6.83%), and groundwater (7.63%). A minor portion of the 16S rRNA gene sequences was retrieved from hypersaline lakes and soils (<5%).

**TABLE 1:** General genomic features of the “*Ca.* Aenigmarchaeota” MAGs reconstructed from hot spring metagenome sequencing

Bins	DRTY-6_1 bin 65	DRTY-6_2 bin 201	DRTY-6_2 bin 202	DRTY-6_2 bin 215	DRTY-7_1 bin 34	GMQ_1 bin 18-1	JZ-2_2 bin 245	QQ_2 bin 128
Genome size (Mbp)	0.75	0.71	0.69	0.55	0.82	0.55	0.52	0.64
GC content (%)	31.6	30.9	28.6	25.7	29.1	25.5	47.9	34.2
*N*_50_	394,136	119,657	15,097	31,689	45,374	62,116	24,348	157,870
No. of scaffolds	19	9	69	25	27	19	25	8
Completeness (%)^*a*^	96.3	92.6	98.1	94.4	90.7	94.4	96.3	94.4
Contamination^*b*^	0	0	0	0	0	0	0	0.93
Strain heterogeneity^*b*^	0	0	0	0	0	0	0	0
No. of RNAs	42	20	35	22	43	47	28	34
5S rRNAs	2	1	1	0	1	1	1	0
16S rRNAs	0	1	1	1	1	1	1	1
23S rRNAs	1	1	0	0	1	1	1	1
tRNAs	39	17	33	21	40	44	25	32
No. of protein-coding genes	865	729	840	662	923	662	637	757
Avg length (bp)	817	857	754	789	820	790	747	747
Coding density (%)	93.9	88.0	92.1	94.5	92.8	94.4	92.3	88.0
Overlapped genes	223 (25.8%)	50 (6.9%)	193 (23%)	113 (17.1%)	229 (24.8%)	202 (30.5%)	105 (16.5%)	154 (20.3%)
No. of genes annotated by COG^*c*^	392 (43.2%)	422 (56.3%)	382 (43.7%)	367 (53.6%)	451 (46.6%)	336 (47.3%)	350 (52.6%)	383 (48.4%)
No. of genes annotated by KO^*c*^	313 (34.5%)	348 (46.4%)	324 (37%)	321 (46.9%)	353 (36.5%)	279 (39.3%)	287 (43.2%)	321 (40.5%)

a Genome completeness was calculated as the percentage of detected marker genes among 54 conserved single-copy genes as listed in Data Set S1 in the supplementary material.

b Genome quality, including contamination and heterogeneity, were estimated by CheckM (16).

c Functional annotation was conducted by uploading genomes to the IMG database.

10.1128/mSystems.00606-21.1 FIG S1 Genomic comparisons of “*Ca.* Aenigmarchaeota” genomes from thermal environments and nonthermal environments. Genome size, GC content, gene number, and average gene length were calculated and visualized using ggplot2 package v3.1.0 in RStudio. The significance tests were conducted using Mann-Whitney *U* test between two groups; *, *P < *0.05; **, *P < *0.01. Download FIG S1, PDF file, 0.8 MB. Copyright © 2021 Li et al. 2021 Li et al. https://creativecommons.org/licenses/by/4.0/ This content is distributed under the terms of the Creative Commons Attribution 4.0 International license.

10.1128/mSystems.00606-21.2 FIG S2 The maximum likelihood-based phylogenetic tree of 16S rRNA gene. 16S rRNA gene sequences within DPANN and TACK superphyla were chosen to construct the phylogenetic tree. The phylogeny was generated using IQ-TREE v1.6.11. Labels in orange show genomes presented in this study. Bootstrap values ≥70 and ≥50 are shown in green and orange dots. Download FIG S2, PDF file, 0.3 MB. Copyright © 2021 Li et al. 2021 Li et al. https://creativecommons.org/licenses/by/4.0/ This content is distributed under the terms of the Creative Commons Attribution 4.0 International license.

10.1128/mSystems.00606-21.6 TABLE S1 Genomic summary of available draft or complete genomes of “*Ca.* Aenigmarchaeota” from public databases. Download Table S1, XLSX file, 0.01 MB. Copyright © 2021 Li et al. 2021 Li et al. https://creativecommons.org/licenses/by/4.0/ This content is distributed under the terms of the Creative Commons Attribution 4.0 International license.

10.1128/mSystems.00606-21.7 DATA SET S1 The occurrences of 54 archaeal conserved single-copy genes in the genomes of “*Ca.* Aenigmarchaeota” and list of genes assigned to metabolic features. Download Data Set S1, XLSX file, 0.1 MB. Copyright © 2021 Li et al. 2021 Li et al. https://creativecommons.org/licenses/by/4.0/ This content is distributed under the terms of the Creative Commons Attribution 4.0 International license.

10.1128/mSystems.00606-21.8 DATA SET S2 Information on 16S rRNA sequences of “*Ca.* Aenigmarchaeota” recruited from the NCBI database. Download Data Set S2, XLSX file, 0.02 MB. Copyright © 2021 Li et al. 2021 Li et al. https://creativecommons.org/licenses/by/4.0/ This content is distributed under the terms of the Creative Commons Attribution 4.0 International license.

### Metabolic features of “*Ca.* Aenigmarchaeota.”

**FIG 2:**
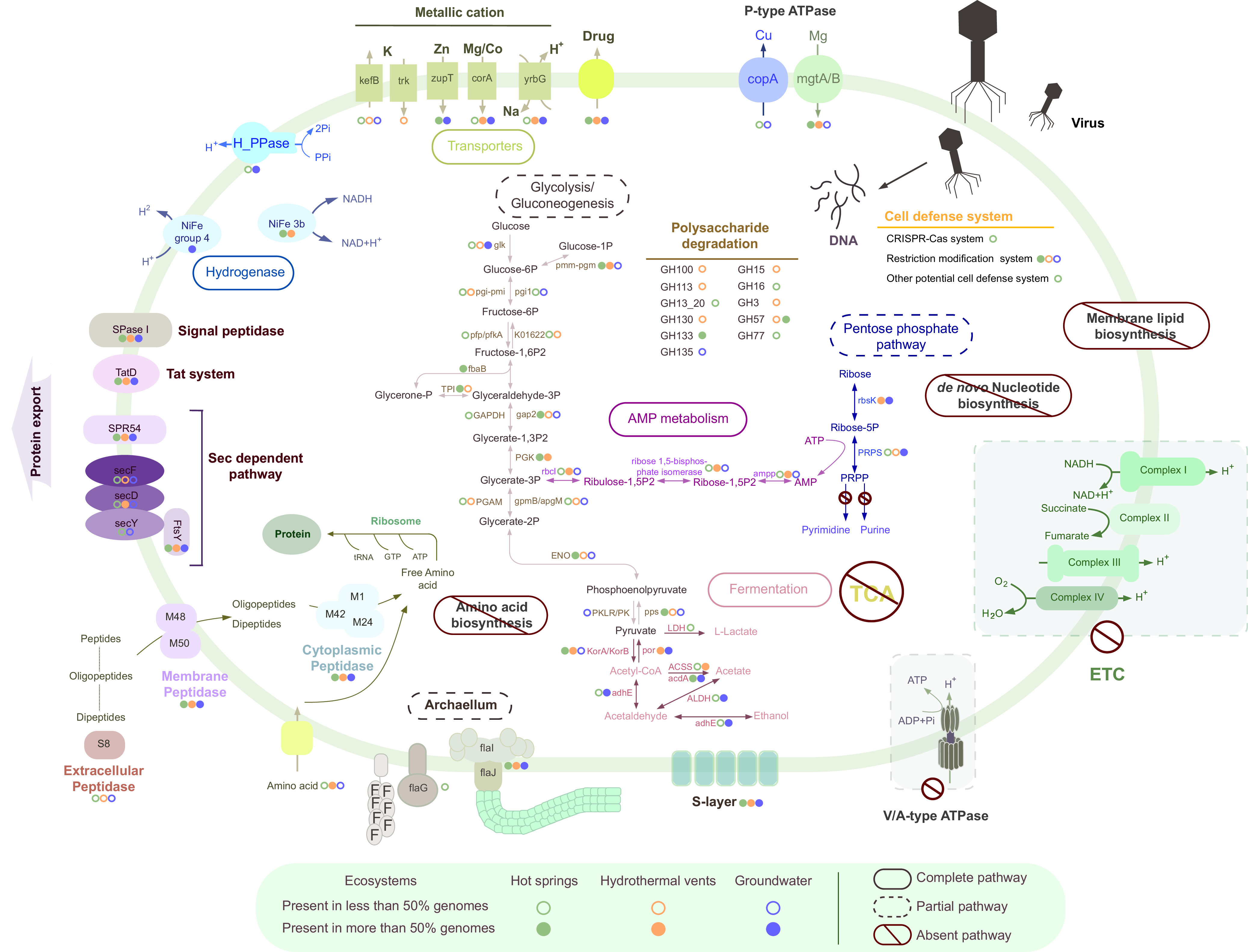
Reconstructed metabolic pathway of “*Ca.* Aenigmarchaeota.” Key genes involved in glycolysis, gluconeogenesis, pentose phosphate pathway, pyruvate metabolism, fermentation, AMP metabolism, protein biosynthesis and exportation, transporters, and flagellum are shown. Solid circles indicate that certain genes are present in more than or equal to 50% of the genomes, while hollow circles are genes presented in less than 50% of the genomes. Abbreviations: TCA, tricarboxylic acid; ETC, electron transport chain; PRPP, phosphoribosyl pyrophosphate.

Based on 8 MAGs from this study and 3 SAGs and 12 MAGs from previous studies, we constructed the metabolic pathways of “*Ca.* Aenigmarchaeota” (Fig. 2). Consistent with previous studies on the DPANN superphylum, all 23 “*Ca.* Aenigmarchaeota” MAGs have limited metabolic capacities. Pathways including the tricarboxylic acid cycle (TCA), fatty acid metabolism, and dissimilatory/assimilatory sulfur and nitrogen metabolism were missing (8, 17, 18). “*Ca.* Aenigmarchaeota” MAGs from hot springs and hydrothermal vents possess an incomplete glycolytic pathway (Fig. 2). All MAGs except DRTY-6_2 bin 201 lack the key enzyme phosphofructokinase (PFK), which impedes the formation of fructose-1,6-bisphosphate (fructose-1,6P) from fructose-6-phosphate (fructose-6P) (Data Set S1). DRTY-6_2 bin 201 seems to have a rather complete glycolysis pathway. However, the lack of glucokinase indicates the incapacity in the production of glucose-6-phosphate (glucose-6P) from glucose. Interestingly, the solely detected glycogen phosphorylase in this MAG and widely distributed phosphoglucomutase suggest that DRTY-6_2 bin 201 can phosphorylate glycogen into glucose-1-phosphate (glucose-1P) and further enter the glycolysis pathway by converting glucose-1P into glucose-6P, which could subsequently enter the rest of glycolysis pathway. The absence of pyruvate kinase (PK) and pyruvate kinase isozymes R/L (PKLR) prohibits the conversion of phosphoenolpyruvate (PEP) to pyruvate during the last step of glycolysis in DRTY-6_2 bin 201. However, phosphoenolpyruvate synthase (*pps*), which might perform the same function as PK in thermophiles (19, 20), was detected in most MAGs in this study, including DRTY-6_2 bin 201. As a result, these genes may provide an alternative glycolysis pathway to DRTY-6_2 bin 201. Subsequently, genes encoding 2-oxoglutarate/2-oxoacid ferredoxin oxidoreductase (*korAB*) in most of the MAGs suggest that “*Ca.* Aenigmarchaeota” is able to catalyze the reaction from pyruvate to acetyl-CoA. The reverse reaction could be performed by pyruvate ferredoxin oxidoreductase (*por*), which is widely detected in MAGs of hydrothermal vents and groundwater. Three MAGs can generate membrane proton motive force (PMF) via a hydrolysis process encoded by the membrane-bound H^+^-phosphatase (H^+^-PPase) (21). Alternatively, PMF could be generated via reactions involved in the degradation of amino acids or Na^+^/H^+^ antiporters (22). However, the absence of the electron transport chain (ETC), especially V/A-type ATPase, may suggest that “*Ca.* Aenigmarchaeota” could not produce ATP via PMF. Each “*Ca.* Aenigmarchaeota” contains at least one type of fermentation pathway. All MAGs from hot springs and groundwater except DRTY-6_2 bin 201 and GMQ_1 bin 18-1 harbor acetate-coenzyme A (acetate-CoA) ligase, which performs the conversion of acetate and ATP from acetyl-CoA, ADP, and phosphate. DRTY-6_2 bin 201, QQ_2 bin 128, and most MAGs from groundwater could utilize *adhE* to produce acetaldehyde and utilize aldehyde dehydrogenase to produce acetate. In addition, QQ_2 bin 128 is predicted to produce lactate and ethanol, based on the presence of l-lactate dehydrogenase, and acetaldehyde dehydrogenase/alcohol dehydrogenase. Moreover, MAGs from hydrothermal vents and DRTY-6_2 bin 201 could utilize acetyl-CoA synthetase (ACSS) to produce acetate as previously described for other DPANN genomes (3). The production of acetate is predicted to support the growth of aerobic/anaerobic respiratory organisms, indicating the role of “*Ca.* Aenigmarchaeota” in the energy cycle of the microbial community (8). These results show that fermentation pathways could be the main sources of ATP generation for “*Ca.* Aenigmarchaeota” (23).

**FIG 3:**
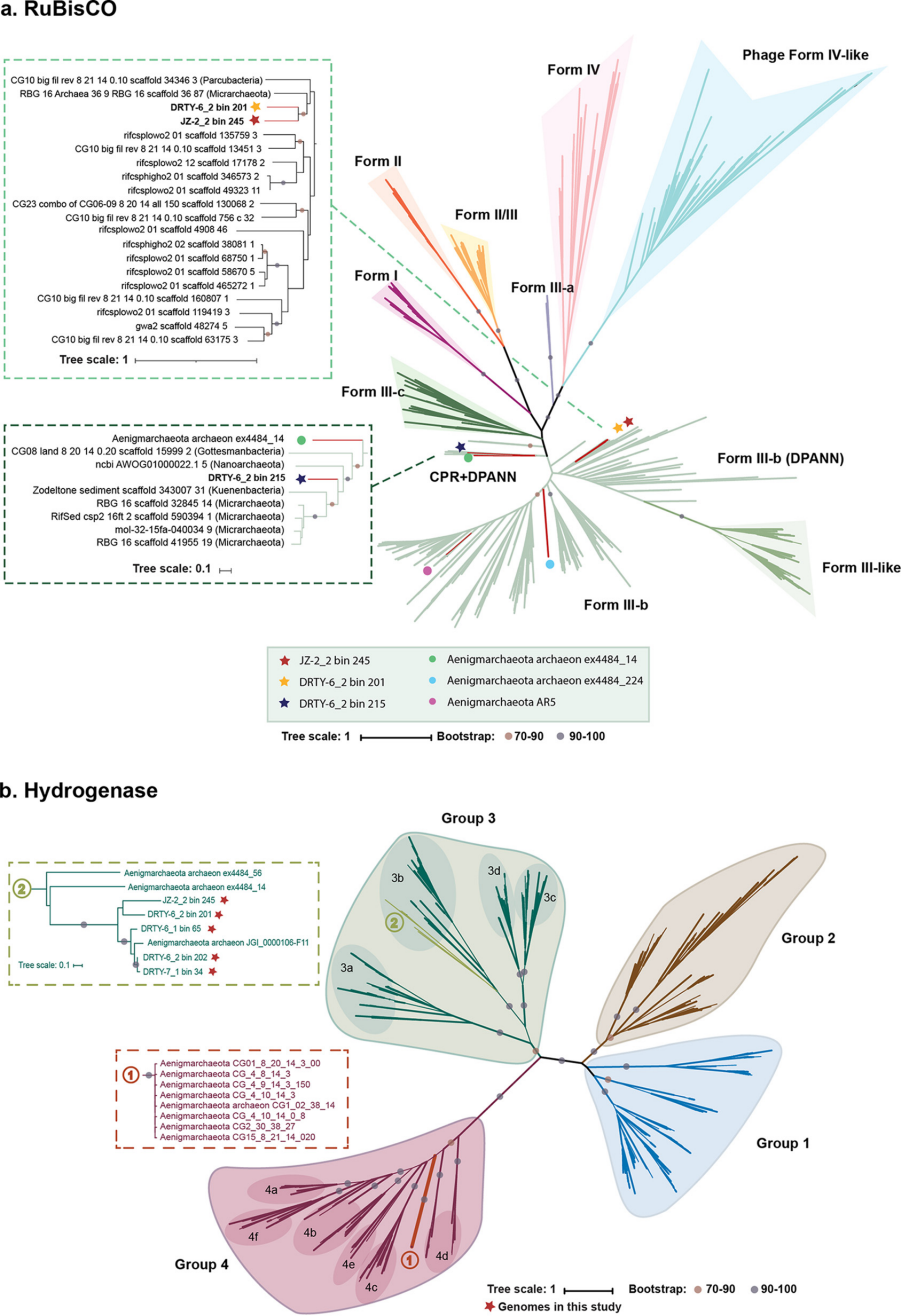
Phylogenetic tree of RuBisCO and hydrogenase. (a) Unrooted maximum likelihood tree of RuBisCO large subunit. RuBisCO genes from the present study are shown in stars. The bootstrap values of main clades in this phylogenetic tree are shown with dots in different colors. (b) Unrooted maximum likelihood tree of hydrogenases. Red stars represent genomes in this study. Dots on the branches indicate the bootstrap values of ≥70. Bootstrap confidences are shown only for the ancestral nodes of each clade.

Two genes relevant to polysaccharide degradation, including α-amylase (for starch and glycogen) and α-1,6-glucosidase (for starch and disaccharides), were identified in many of the hot spring MAGs (Data Set S1). DRTY-6_1 bin 65 might also degrade and utilize pullulan (GH13_20) and xyloglucan (GH16) (24, 25). Aside from α-amylase, MAGs from hydrothermal vents harbored different and more glycoside hydrolases, including β-glucosidases (for disaccharides), glucoamylases (for starch), β-1,2-mannosidases (for β-1,2-mannotriose and β-1,2-mannobiose), and endo-1,4-β-mannanase (for β-1,4-mannans, β-1,4-galactomannans, and β-1,4-glucomannans). However, MAGs from groundwater only had α-1,4-galactosaminogalactan hydrolase (for galactosaminogalactan). This might reflect a more active carbohydrate metabolism in thermal environments. None of the known carbon fixation pathways were detected in these MAGs, though three MAGs contain archaeal ribulose-bisphosphate carboxylase (RuBisCO). As previously described in other archaea, RuBisCO genes may function in the CO_2_-incorporating AMP pathway, together with genes encoding AMP phosphorylase and ribose-1,5-biphosphate isomerase (26, 27). This pathway could produce glycerate-3-phosphate that enters the glycolysis pathway. Phylogenetic analysis suggests that the six RuBisCO genes recovered from “*Ca.* Aenigmarchaeota” belong to the form III group, of which five are from thermal environments (Fig. 3a). Four of them belong to form III-b, and the remaining two could be classified as a novel lineage that clustered with RuBisCO genes from candidate phyla radiation (CPR) genomes, which were previously suggested to have been passed by HGT from “*Ca.* Aenigmarchaeota” to CPR (26). Additionally, five MAGs distributed in both thermal and nonthermal ecosystems were identified to encompass all three genes involved in the AMP pathway (Data Set S1). We also identified different types of hydrogenases in “*Ca.* Aenigmarchaeota.” Eight MAGs from thermal environments harbor NiFe 3b-type hydrogenases, which are clustered into one clade (Fig. 3b). This type of hydrogenase is functionally reversible and is capable of catalyzing the oxidation for anabolic metabolism or evolution of H_2_ during fermentation (28). Unlike the MAGs from thermal environments, NiFe 3b-type of hydrogenases are absent in nonthermal environments. Instead, membrane-bound hydrogenases (group 4) are identified, illustrating that different strategies are used by nonthermophiles in producing PMF and H_2_.

Despite the possession of genes involved in glycolysis and the fermentation pathway, the absence of many pivotal pathways strongly suggests a symbiotic lifestyle for “*Ca.* Aenigmarchaeota.” First, this archaeal phylum is devoid of *de novo* amino acid biosynthetic pathways. Although we found a variety of extracellular peptidases, membrane peptidases, and cytoplasmic peptidases that can degrade extracellular and intracellular proteins and peptides (Data Set S1) (3), only a few amino acid transporters were detected. The only identified one is an uncharacterized amino acid transporter (arCOG00009), suggesting a poor ability in the transport of peptides/amino acids extracellularly. Therefore, “*Ca.* Aenigmarchaeota” presumably obtain amino acids from hosts by physical contact, similar to *Nanohaloarchaeota* and *Nanoarchaeota* (29–31), and rely on a great number of peptidases to recycle their amino acids. Second, *de novo* nucleotide biosynthetic pathways are absent in most of the genomes of this phylum (32). Moreover, genes for purine and pyrimidine salvage pathways are rarely detected in most MAGs from hot springs (Data Set S1), illustrating the further reliance on a host to provide requisite nutrients. Third, “*Ca.* Aenigmarchaeota” genomes are unable to synthesize cell membranes *de novo* due to the lack of genes for synthesis of sterol isoprenoids involved in the mevalonate pathway (MVA), although genes encoding mevalonate kinase, glycerol-1-phosphate dehydrogenase, and associated enzymes for phospholipid biosynthesis have been detected (33, 34).

Cell-surface structures might enable the interactions between DPANN archaea with their hosts (30). Genes encoding S-layers, a subset of confirmed archaellum proteins (FlaG, FlaI, and FlaJ), and several adjacent archaellum homologs are identified in most “*Ca.* Aenigmarchaeota” MAGs. Notably, type-IV pili in “*Ca.* Aenigmarchaeota” are solely found from MAGs inhabiting thermal environments (35–37). To a certain extent, these genes endow “*Ca.* Aenigmarchaeota” with protection, motility, and cell-to-cell attachment abilities, which might consequently facilitate host-symbiont interactions.

### Stress responses used by “*Ca.* Aenigmarchaeota.”

**FIG 4:**
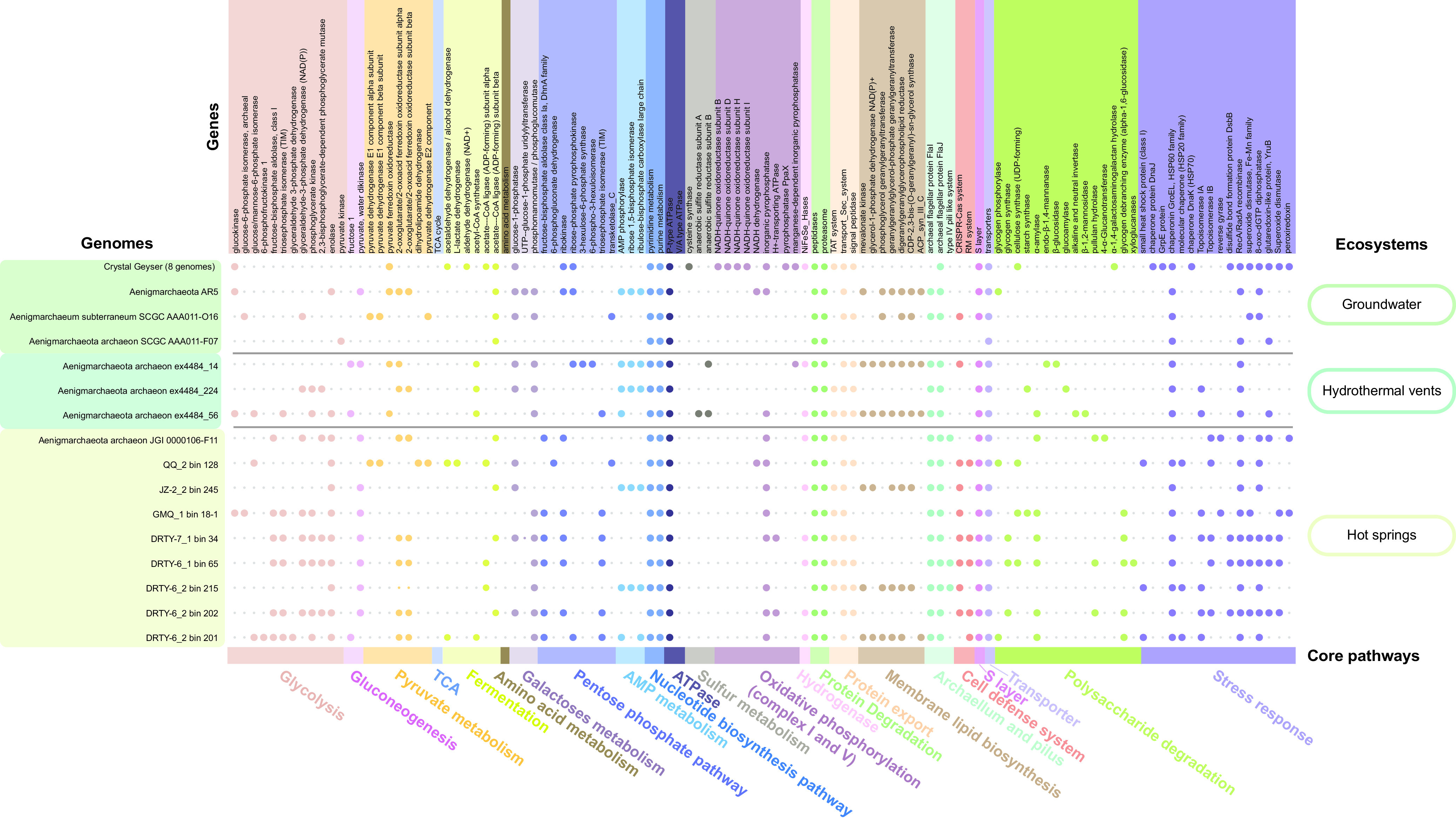
The core metabolic pathways with the presence/absence of genes of all “*Ca.* Aenigmarchaeota” genomes. The metabolic potential (columns) is mainly generated based on KEGG orthologs (KOs), clusters of orthologous groups (COGs), and archaeal clusters of orthologous groups (arCOGs). Genomes (rows) of “*Ca.* Aenigmarchaeota” were clustered by ecosystems. Eight genomes obtained from groundwater in Crestal Geyser were shown as one row because of the high metabolic similarity of the eight genomes. Small gray dots represent genes or metabolic pathways that were absent, and big dots represent genes or metabolic pathways that were present. Distinct metabolic pathways were distinguished by different colored dots. See Data Set S1 in the supplemental material for details.

Comparative genomics showed that “*Ca.* Aenigmarchaeota” inhabiting thermal environments harbor higher abundances of genes involved in genetic information processing, including “transcription,” “translation,” “replication and repair,” and “folding, sorting, and degradation” (Fig. S3a). In addition, genes involved in “cell motility” and “posttranslational modification, protein turnover, and chaperones” predominantly were enriched in thermophiles (Fig. S3b). This reflects the fact that cells at high temperatures have to combat constant thermal denaturation of both macromolecules and monomers (38–40). “*Ca.* Aenigmarchaeota” MAGs from thermal environments have evolved multiple strategies to overcome this stress. Chaperonin GroEL, associated with the repair of DNA and protein damage caused by high temperature, was present in all “*Ca.* Aenigmarchaeota” genomes (Fig. 4) (8, 41). The prevalence of DNA repair protein RadA could be used for homologous recombination and as an alternative strategy for DNA repair (42), indicating the pervasiveness of genome reduction among these genomes. Type I (IA- and IB-type) topoisomerases and reverse gyrases, the latter considered a hallmark of hyperthermophily, were solely detected in MAGs from thermal environments, which could stabilize DNA and modulate DNA topology to maintain the structure and integrity of chromosomes (40, 43, 44). HSP70 (DnaK), DnaJ, and GrpE were absent in MAGs from thermal environments but were commonly detected in groundwater MAGs (Fig. 4), which is consistent with the potential role of this system in the adaptation to mesophily (45).

10.1128/mSystems.00606-21.3 FIG S3 Metabolic category of genomes obtained from three ecosystems. Based on annotation results of KOs (a) and arCOGs (b), relative abundance of each metabolic category of three ecosystems was represented by bars with different fillings. To avoid biases caused by the poor quality of reconstructed genomes, three genomes of single-cell sequencing were discarded. A higher-level metabolic category was shown by different color bars. Error bars represent standard errors. Differences among three ecosystem types in all categories were assessed using analysis of the variance (ANOVA; function aov in R package “agricolae”), followed by least significant different (LSD) *post hoc* all-pairwise comparisons test (function LSD.test in R package “agricolae”). Significant differences among groups were marked with letters on the tops of the bars. Bars that do not share the same letters (such as “a” and “b”) suggest that there is a significant difference between them. Otherwise, no significant differences were observed. Genes significantly enriched in hot springs were marked with star-shaped symbols. Download FIG S3, PDF file, 0.9 MB. Copyright © 2021 Li et al. 2021 Li et al. https://creativecommons.org/licenses/by/4.0/ This content is distributed under the terms of the Creative Commons Attribution 4.0 International license.

The presence of cell defense systems protects prokaryotic cells from virus infection. Based on the results of functional annotation, we investigated cell defense systems in “*Ca.* Aenigmarchaeota.” DRTY-7_1 bin 34 was detected to contain a CRISPR-Cas system (Class III-A; Fig. S4a). All three types of restriction modification (RM) systems were found in “*Ca.* Aenigmarchaeota” (46) (Fig. S4b). The most frequently detected type III RM system was found in 15 (65%) of the total MAGs. Five of the eight MAGs from this study harbor a type II RM system, indicating an alternative common strategy for terrestrial thermal microbes to resist virus infection. Additionally, the recently discovered Hachiman system was detected in DRTY-6 bin 65 and the Gabija system was detected in DRTY-6 bin 215, providing broad protection against viruses (47) (Fig. S4c). The wide distribution of defense systems in thermophilic “*Ca.* Aenigmarchaeota” suggests that viruses could be an important threat to the survival of microbes in hot spring ecosystems (48–50). Viruses with different morphologies have been detected in hot springs and are highly active *in situ* (14, 47). Hence, it seems plausible that “*Ca.* Aenigmarchaeota” may confer their hosts with immunity to viruses by serving as “viral decoys” (51). The attracted virus could be degraded, and the released DNA could be recycled as a nucleotide source (51). Therefore, the host-symbiont interaction between “*Ca.* Aenigmarchaeota” and its potential hosts appear to be mutually beneficial.

10.1128/mSystems.00606-21.4 FIG S4 Cell defense systems in “*Ca.* Aenigmarchaeota.” Different strategies, including the CRISPR-Cas system (a), restriction modification (RM) systems (b), and other potential cell defense systems (c), were used by “*Ca.* Aenigmarchaeota” to defend against virus infection. The gray arrays in (c) represent hypothetical proteins. Download FIG S4, PDF file, 0.8 MB. Copyright © 2021 Li et al. 2021 Li et al. https://creativecommons.org/licenses/by/4.0/ This content is distributed under the terms of the Creative Commons Attribution 4.0 International license.

### Horizontal gene transfer in “*Ca.* Aenigmarchaeota.”

**FIG 5:**
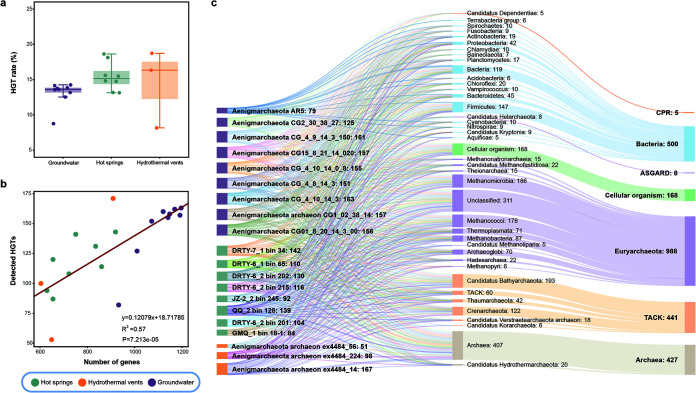
The identified horizontally gene transfers (HGTs) in genomes from this study, hydrothermal vents, and groundwater. (a) Comparisons of HGT rates between these three groups. (b) A linear regression analysis was conducted to model the relationship between genomes size and detected interphylum HGTs. The fitted formula was shown on the plot with *R*^2^ = 0.57 and *P = *7.213E−05. (c) The Sankey plot shows the detected interphylum HGTs and potential donors for each genome. Number of donors that were below five were removed. Interphylum HGTs were detected using HGTector as described in Materials and Methods.

Horizontal gene transfer has been recognized as a substantial force in shaping the genetic diversity of prokaryotes (52–55). To ensure a high-quality detection of HGTs, we removed three low-quality genomes for HGT analysis (see details in Materials and Methods). Surprisingly, results uncovered a lower proportion of HGT-derived genes in “*Ca.* Aenigmarchaeota” than in thermophilic, free-living archaea, for example, *Aigarchaeota* (mean 14.2% versus 22.9%; Mann-Whitney *U* test, *P = *8.687E−06) (7). Intriguingly, “*Ca.* Aenigmarchaeota” MAGs from different ecosystems possessed comparable percentages of HGT-derived genes (Fig. 5a), in which significant positive correlation (Pearson’s *R*^2^ = 0.57, *P = *7.213E−05) was observed between detected HGTs and predicted gene totals in corresponding genomes regardless of ecosystems and genome sizes (Fig. 5b). By looking into the potential donors, we found that members from *Euryarchaeota* (998, 38.7%), “*Ca.* Bathyarchaeota” (193, 7.49%), and *Firmicutes* (147, 5.70%) are the top three contributors to the genetic innovations of “*Ca.* Aenigmarchaeota” MAGs (Fig. 5c and Data Set S3). Among the HGTs from *Euryarchaeota*, 186 (7.2% among all HGTs) are derived from *Methanomicrobia* and 178 (6.9%) are from *Methanococcus*. The high percentage of HGTs derived from *Euryarchaeota*, *Crenarchaeota*, and “*Ca.* Bathyarchaeota” is consistent with previous studies reporting of symbioses between DPANN and TACK and *Euryarchaeota* (10, 29, 30, 56).

10.1128/mSystems.00606-21.9 DATA SET S3 Identified potential horizontal transferred genes and donors of HGTs in “*Ca.* Aenigmarchaeota” MAGs. Download Data Set S3, XLSX file, 0.3 MB. Copyright © 2021 Li et al. 2021 Li et al. https://creativecommons.org/licenses/by/4.0/ This content is distributed under the terms of the Creative Commons Attribution 4.0 International license.

To reveal potential factors that facilitate the adaptation of “*Ca.* Aenigmarchaeota” across groundwater, hot springs, and hydrothermal vents, we compared the acquired genes between these groups. Results indicated very little overlap between HGT-derived genes among these three ecosystems. Only 54 KEGG orthologs (KOs) and 67 archaeal clusters of orthologous groups (arCOGs) (11.04% and 12.27% among all KO and arCOG assignable HGTs) are shared by all the three groups (Data Set S3), suggesting that they have distinct adaptation strategies.

Through the BLAST-based analysis, as well as phylogenetic analysis, we can verify that HGT plays a crucial role during the adaptation to different stresses. For instance, the detected reverse gyrases in “*Ca.* Aenigmarchaeota” genomes seem to derive from “*Ca*. Bathyarchaeota,” which may have improved the fitness of these organisms to inhabit high-temperature environments (Fig. S5a). Also, several genes encoding superoxide dismutase (SOD2) (Fig. S5b) and 8-oxo-dGTP diphosphatase (*mutT*) (Fig. S5c) were identified as HGTs, which could be used to resist oxidative damage and to generate PMF (22). Two genes, including transcription initiation factor IIB (TFIIB) and phage integrase, in these two MAGs DRTY-6_1 bin 65 and DRTY-7_1 bin 34 were identified as belonging to phyla outside DPANN, both of which show high identity to *Euryarchaeota*. However, neither of them was identified as HGTs by HGTector. From the constructed phylogeny, the TFIIB gene in *Theionarchaea* archaeon DG-70 is surrounded by genes from “*Ca.* Aenigmarchaeota,” suggesting that members of “*Ca.* Aenigmarchaeota” are possible gene donors rather than recipients (Fig. S5d). In DRTY-6 bin 65, both genes are in the same scaffold. The taxonomic information of genes close to TFIIB is mostly affiliated with “*Ca.* Aenigmarchaeota,” consolidating the inference of vertical inheritance of this gene. The phage integrase may be horizontally transferred from *Euryarchaeota*, since 6 of the 10 downstream genes are close relatives to *Theionarchaea* (51.3 to 77.4%) and three are close relatives to *Thermoplasmata* (48.3 to 61.1%). Among them, three genes exhibit homologies to methyltransferase, DNA-binding protein, and restriction endonucleases associated with type II restriction modification (RM) systems. This suggests that integrase-mediated HGT may confer “*Ca.* Aenigmarchaeota” the special niche to resist virus infection. Overall, the above observations further support that HGT plays a substantial role in shaping the genetic diversity of “*Ca.* Aenigmarchaeota” for stress response.

10.1128/mSystems.00606-21.5 FIG S5 Phylogenetic trees of four important horizontally transferred genes of “*Ca.* Aenigmarchaeota.” (a) Phylogenetic tree of reverse gyrase (*rgy*). The phylogenetic tree was rooted using MAD v2.2 with the default parameters. (b) Phylogenetic tree of superoxide dismutase (SOD2). (c) Phylogenetic tree of 8-oxo-dGTP diphosphatase (*mutT*). (d) Phylogenetic tree of transcription initiation factor IIB (TFIIB). Labels in red stars are from “*Ca.* Aenigmarchaeota” MAGs in this study. Download FIG S5, PDF file, 1.3 MB. Copyright © 2021 Li et al. 2021 Li et al. https://creativecommons.org/licenses/by/4.0/ This content is distributed under the terms of the Creative Commons Attribution 4.0 International license.

### Putative functional and genetic interaction partners of “*Ca.* Aenigmarchaeota” inferred from *in situ* communities.

Cell-to-cell contact possibly leads to an opportunity for extensive HGTs (57–59). The inferred HGTs of “*Ca.* Aenigmarchaeota” may facilitate us to infer their potential hosts. However, only xenologous sequences with high identities, the so-called “recent HGTs,” could be used for the inference of current symbiotic relationships between the associated donor and recipient (59). Therefore, we ruled out the possibilities of Bacteria as potential hosts since most of the transferred genes represent ancient events with high divergence, though some Bacteria may contribute a lot to the genomic innovation of “*Ca.* Aenigmarchaeota.” For instance, those genes transferred from *Firmicutes* only shared ∼42% of the identities to the recipient genes in “*Ca.* Aenigmarchaeota.” Additionally, most DPANN, such as *Nanoarchaeota*, *Nanohaloarchaeota*, and “*Ca*. Micrarchaeota,” are incapable of synthesizing membrane spontaneously and must rely on assistance from their hosts (8, 30, 60, 61). Therefore, it is more likely that the putative hosts are restricted to Archaea due to the similar membrane structure shared by DPANN and most other archaea (8). Additionally, all previous studies support our conjecture that DPANN archaea were exclusively associated with archaea to form the symbiotic relationship (e.g., Nanoarchaeum equitans and Ignicoccus hospitalis, “*Ca.* Micrarchaeota acidiphilum” Mia14 and Cuniculiplasma divulgatum PM4, “*Ca.* Nanohaloarchaeota antarcticus” and *Halorubrum lacusprofundi*, “*Ca.* Huberiarchaeum crystalense,” and “*Ca*. Altiarchaeum hamiconexum”) (17, 29–31, 62, 63).

**FIG 6:**
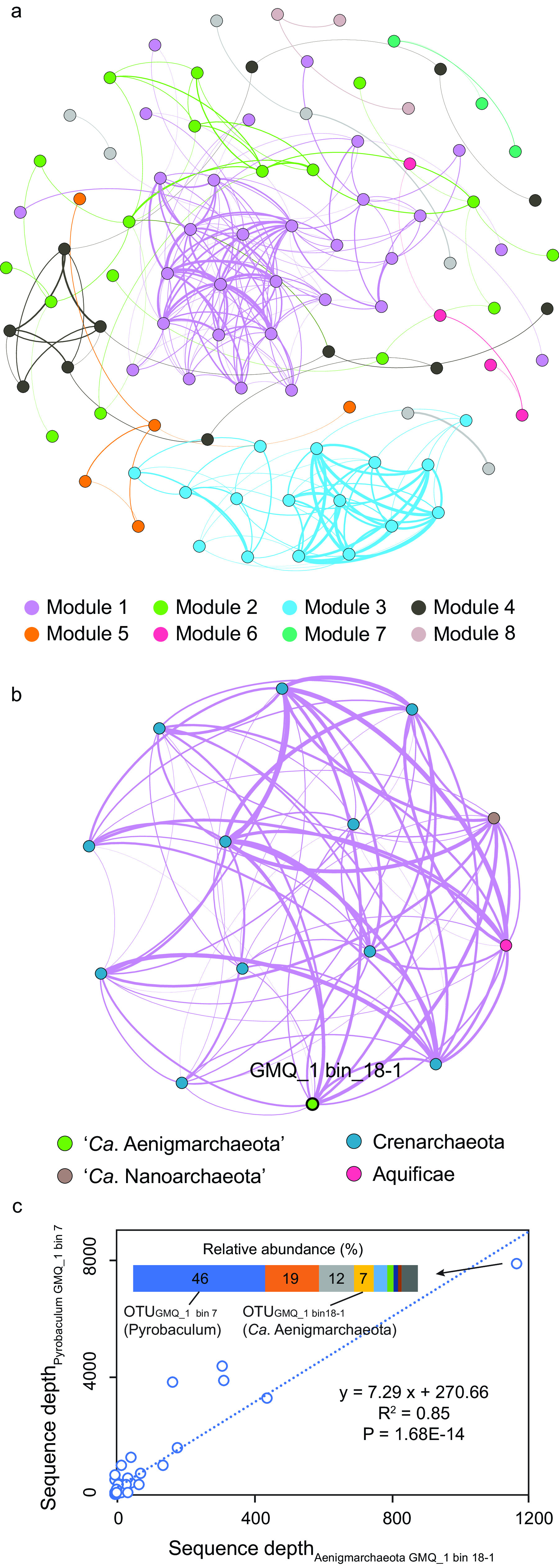
The co-occurrence network between “*Ca.* Aenigmarchaeota” and other community members. (a) The co-occurrence network was constructed based on the depth of the representative sequences of all OTUs in all samples using SparCC (95) with the default parameters, and 100 bootstrap samples were used to infer pseudo-*P* values. The nodes represent OTUs at a 95% cutoff, and these edges denote significant (*P < *0.05, two-sided) and robust correlations (rho > 0.6) between pairwise OTUs. OTUs are colored by modularity classes. Nodes have the same size, and edges have the same thickness. (b) The subnetworks that contain “*Ca.* Aenigmarchaeota” *rpS3* genes. Only nodes and edges that have connections with “*Ca.* Aenigmarchaeota” in the corresponding modules are shown. Modules 1 in (a) were detected to contain “*Ca.* Aenigmarchaeota” *rpS3* genes. The size of each node is proportional to the number of connections (i.e., degree). OTUs are colored by the phylum-level taxonomy. The thickness of edges denotes the Spearman rank correlation coefficients (rho values). Edges in purple show the connections between “*Ca.* Aenigmarchaeota” (green circles deviated from core networks) and other members. (c) Correlation between *Pyrobaculum* GMQ_1 bin 7- and GMQ_1 bin 18-1-associated OTUs. The two OTUs are observed in 33 out of 88 metagenomic samples. Results show a statistically significant positive correlation (Pearson’s *R*^2^ = 0.85, *P < *1.68E−14) between the sequence depths of these two OTUs.

To further explore the potential host-symbiont relationships between “*Ca.* Aenigmarchaeota” and other archaea, a network interface of microbial communities in hot springs was constructed (see Materials and Methods), aiming to reveal microbial co-occurrence patterns and possible ecological interactions (59, 62–65). The network encompasses 97 nodes with 257 edges, and only one of them is identified as “*Ca.* Aenigmarchaeota” (Fig. 6a). After extracting the module that contains an “*Ca.* Aenigmarchaeota” operational taxonomic unit (OTU) (GMQ-1 bin_18-1), we observed tight connections (|rho| ≥ 0.6 and pseudo-*P* values of <0.05) between GMQ-1 bin_18-1 and 11 OTUs from *Crenarchaeota* (rho values are listed in Data Set S4; Fig. 6b).

10.1128/mSystems.00606-21.10 DATA SET S4 Putative interaction partners of “*Ca.* Aenigmarchaeota” inferred from recent horizontal gene transfer (HGT), co-occurrence network analyses, and detected recent HGTs from the corresponding communities and OTU table of 88 hot spring samples. Download Data Set S4, XLSX file, 0.4 MB. Copyright © 2021 Li et al. 2021 Li et al. https://creativecommons.org/licenses/by/4.0/ This content is distributed under the terms of the Creative Commons Attribution 4.0 International license.

**FIG 7:**
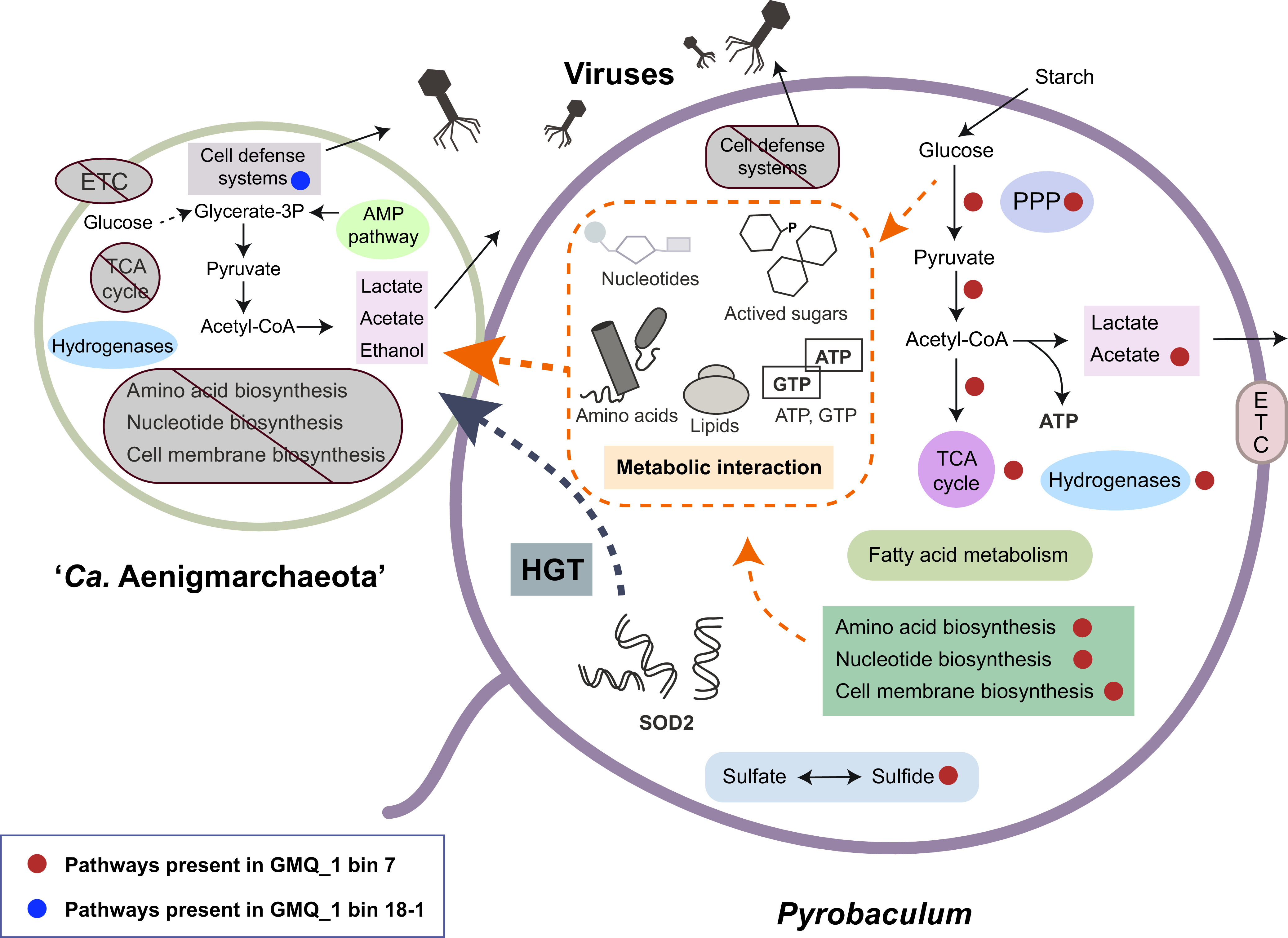
The potential interaction between “*Ca.* Aenigmarchaeota” and *Pyrobaculum*. Based on the annotation results of eight MAGs of “*Ca.* Aenigmarchaeota” in this study and the metabolic features of *Pyrobaculum* GMQ_1 bin 7 and other *Pyrobaculum* genomes, the schematic metabolic capacities and possible interaction scenario of “*Ca.* Aenigmarchaeota” and *Pyrobaculum* were shown. Metabolic pathways that presented in GMQ_1 bin 18-1 and GMQ_1 bin 7 were marked with blue and red circles, respectively. Abbreviations: TCA cycle, tricarboxylic acid cycle; ETC, electron transport chain; PPP, pentose phosphate pathway.

Notably, we identified a recent HGT event between “*Ca.* Aenigmarchaeota” and one of the *Crenarchaeota* OTUs, which strengthened the putative symbiont-host relationship between them. The SOD2 gene in GMQ_1 bin 18-1 shows 89.1% identity and 100% of coverage to the gene in *Pyrobaculum* sp. WP30. By looking into the belonging sample, we identified a SOD2 gene from the GMQ_1 bin 7 that shares a high identity (89%) to the one in GMQ_1 bin 18-1. Remarkably, the GMQ_1 bin 7 MAG could be assigned to the genus *Pyrobaculum* as well. BLAST searches suggest that all SOD2 genes except the one in GMQ_1 bin 18-1 show identities of <30%, indicating the recent HGT event specifically occurs in GMQ_1 bin 18-1 rather than in all “*Ca.* Aenigmarchaeota” members. In addition, the OTUs that GMQ_1 bin 7 and GMQ_1 bin 18-1 belonged to have a statistically significant positive correlation (Pearson’s *R*^2^ = 0.85, *P < *1.68E−14; Fig. 6c). These two bins are the first and fourth most abundant organisms in one sample by taking up greater than 50% of the cells in total. Based on the metabolic features of “*Ca.* Aenigmarchaeota” and *Pyrobaculum*, we proposed the potential interaction scenario between them (Fig. 7) (66). Specifically, for the MAGs of this study, *Pyrobaculum* GMQ_1 bin 7 could provide amino acids, nucleotides, membrane lipids, ATP, and active sugars to support growth of GMQ_1 bin 18-1, and GMQ_1 bin 18-1 possessed RM systems to protect the host for cell defense, which were absent in GMQ_1 bin 7. Additionally, the recent HGT of the SOD2 gene from GMQ_1 bin 7 provides GMQ_1 bin 18-1 with the capacity of oxidative stress resistance. However, the *Pyrobaculum* OTU is completely absent in the five communities where other “*Ca.* Aenigmarchaeota” MAGs in this study came from (Data Set S4). These results indicate that “*Ca.* Aenigmarchaeota” microbes in this study are likely to be associated with different interaction partners.

### Conclusions.

The enigmatic “*Ca.* Aenigmarchaeota” is still underexplored due to the insufficient cultured representatives or assembled genomes available. Here, we expanded the phylogenetic diversity of “*Ca.* Aenigmarchaeota” in hot spring environments and showed that “*Ca.* Aenigmarchaeota” can be found in diverse ecosystems on earth. They harbor limited metabolic capacities by missing several pivotal biosynthetic pathways, such as nucleotide, amino acid, and cell membrane biosynthesis, suggesting that such molecules need to be obtained from the environment or from the host as symbionts. Comparative genomics analysis reveals that genomes from thermal environments possess smaller genome sizes but stronger capacities in metabolizing carbohydrates. HGT identifies a salient number of gene flows from TACK and *Euryarchaeota* to “*Ca.* Aenigmarchaeota,” especially the genes related to stress responses. By conducting co-occurrence network and recent HGT detection analyses, we highlight the power of informatics analysis in identifying putative interaction partners. However, even though significant correlations and genetic interactions are observed, further analyses such as fluorescence *in situ* hybridization, should be integrated to confirm the inference of host-symbiont relationship. Overall, this study enables us to better understand the metabolic potentials and possible interactions between “*Ca.* Aenigmarchaeota” and their putative hosts, shedding light on the understanding of coevolution between hosts and symbionts.

## MATERIALS AND METHODS

### Site description, sampling, DNA extraction, and sequencing.

All five hot spring sediment samples were collected from Tengchong County, Yunnan, China (24.95, 98.44) in January 2016 and May 2017. DRTY-6_1, DRTY-7_1, and GMQ_1 samples were collected from DiReTiYanQu (DRTY), GuMingQuan (GMQ), and QiaoQuan (QQ) hot springs in Rehai National Park in January 2016. DRTY-6_2 and QQ_2 samples were collected from DiReTiYanQu and QiaoQuan (QQ) hot springs in May 2017. JZ-2_2 was collected from the JinZe Hot Spring Resort in May 2017. DiReTiYanQu is an artificial concrete hot spring landscape experiencing area. DRTY-6 and DRTY-7 are two medium-size pools that have mixtures of spall, sand, and soil on the bottom. The pH and temperatures of DRTY-6 were 6.1/50°C in January 2016 and were 6.0/60°C in May 2017. The pH and temperature of DRTY-7 were 6.0 and 56°C, respectively, in January 2016. GuMingQuan (GMQ) is a pool with a hot spring fall above and with a length, width, and depth of around 98, 79, and 9.5 cm, respectively. Leaf litter and other debris on the bottom could be seen clearly. The sampling site is located upstream of the GMQ pool, which was named GMQS. The pH and temperature of GMQ were 9.0 and 89°C, respectively, in January 2016. QiaoQuan (QQ) is a hot spring stream flowing out from a soil slope with a rust-color trace. This spring is surrounded by bush and grass. The pH and temperature of QQ were 7.2 and 77°C, respectively, in May 2017. The JinZe-2 (JZ-2) pool is an artificial concrete cubic hot spring water reservoir with a ceiling covering the top in JinZe Hot Spring Resort. The JZ-2 pool contains turbid water and sediments on the bottom of the pool. The pH and temperature of JZ-2 were 7.6 and 63°C, respectively, in May 2017. The top 1 cm of sediment of each site was collected with a sterile iron spoon and transferred to a 50-ml centrifuge tube. All sediment samples were then stored in liquid nitrogen before transporting to the lab and were stored at −20°C in the lab until DNA extraction.

Community DNA was extracted from approximately 20 g of sediment for each sample using the PowerSoil DNA isolation kit (MoBio). Libraries with an insert size of 350 bp were constructed by using an M220 Focused-ultrasonicator NEBNext and an Ultra II DNA library prep kit. The concentration of genomic DNA was measured with a Qubit fluorometer. The total genomic DNA was sequenced using an Illumina Hiseq 4000 instrument at Beijing Novogene Bioinformatics Technology Co., Ltd. (Beijing, China). On average, 30 giga base pairs (Gbp) (2 × 150 bp) of raw sequencing data for each sample were generated.

### Metagenomic assembly and genome binning.

Raw sequencing data were preprocessed to eliminate replicated reads and trim bases with low qualities, following workflows that were described previously (67). All quality reads of each data set were *de novo* assembled using SPAdes v3.9.0 (68) with the following parameters: -k 33,55,77,99,111 –meta. Scaffolds with a length of <2,500 bp in each assembly were removed. BBMap v38.85 (http://sourceforge.net/projects/bbmap/) with the parameters k = 15 minid = 0.97 build = 1 was used to compute the coverage information by mapping clean reads to the corresponding assembled scaffolds without cross-mapping. Genome binning was performed based on the calculated sequence depth and tetranucleotide frequency (TNF) of each scaffold using MetaBAT v2.12.1 (69). Marker genes that occurred more than once in each bin were treated as contaminations, and associated contigs were removed manually. Specifically, genome bins were visualized by fragmenting each scaffold into pieces with the length ranging from 5 to 10 kb using ESOM v1.1 (70) for further curation, in which the discordant points were removed manually from the clusters. Scaffolds with similar TNFs but abnormal sequence depth (the abnormal sequences depth were examined manually, and the difference was mostly over 10-fold) compared to other scaffolds in the corresponding bins were also discarded. Subsequently, quality reads of the associated samples of each optimized genome bins were recruited by mapping onto all optimized genome bins using BBMap and were reassembled using SPAdes under the “–careful” mode with the parameter “-k 21,33,55,77,99,127.” Contaminations and strain heterogeneity were estimated by CheckM v1.0.12 (71); genome completeness was estimated by calculating the proportion of detected marker genes among 54 conserved archaeal single-copy genes (SCGs) (Data Set S1) (3). Finally, eight MAGs identified as “*Ca.* Aenigmarchaeota” were kept for the later analysis.

### Functional annotation of “*Ca.* Aenigmarchaeota” genomes.

The eight MAGs were submitted to the Integrated Microbial Genomes & Microbiomes (IMG-M) (https://img.jgi.doe.gov/cgi-bin/m/main.cgi) database for gene prediction and functional annotation. For comparative genomics analysis, the annotation pipeline was also conducted locally. In brief, putative protein-coding sequences (CDS) of all MAGs, including eight MAGs from the present study and 15 genomes downloaded from public databases, were determined using Prodigal v2.6.3 (72) under the “-p single” model. Functional annotations were performed by comparing predicted CDSs against KEGG (73), evolutionary genealogy of genes: nonsupervised orthologous groups (eggNOG) (74), Pfam (75), and arCOG (76) databases using DIAMOND v0.7.9 (77) with a cutoff E value of <1E−5. rRNA-coding regions were identified using RNAmmer v1.2 (78). All MAGs were uploaded to the web server of tRNAscan-SE v2.0 (79) to identify the tRNA. The dbCAN2 webserver (80) was used to identify carbohydrate-active enzymes based on the carbohydrate-active enzymes (CAZy) database. The localization signals of genes annotated as peptidases were determined using the online tool PSORTb v3.0 (81). To detect the putative CRISPR-Cas systems in “*Ca.* Aenigmarchaeota” MAGs, tandem repeats, and spacers were identified using the online tool CRISPRFinder (82). Then, the genes nearby the region (both the upstream and downstream) were investigated manually to decide the type. The restriction modification (RM) systems and other cell defense systems, including Hachiman and Gabija reported in a previous study (44), were identified according to the KEGG and arCOG annotation results.

### Phylogenetic and phylogenomic analysis.

Sixteen ribosomal protein sequences (L2, L3, L4, L5, L6, L14, L15, L16, L18, L22, L24, S3, S8, S10, S17, and S19) were selected to reconstruct the phylogenomic tree (83). These sequences were identified by AMPHORA2 (84) and aligned using MUSCLE v3.8.31 with 100 iterations (85). The poorly aligned regions were eliminated using TrimAl v1.4.rev22 (86) with the parameters set as -gt 0.95 -cons 50. Then, multiple alignments were concatenated and applied to reconstruct a maximal likelihood phylogenetic tree using IQ-TREE v1.6.11 with the following parameters: iqtree -s a -alrt 1000 -bb 1000 -nt AUTO (87, 88).

16S rRNA genes were predicted for each MAG using RNAmmer. MAGs without 16S rRNA genes were further searched against the Ribosomal Database Project (RDP) database (downloaded on 18 October 2018) (89) using the BLASTn v2.8.1+ program, and sequences with a length of >300 bp were selected and combined with those retrieved by RNAmmer. All 16S rRNA sequences were aligned using the online tool SINA v1.2.11 (90). Columns containing more than 95% gaps were removed, after which a maximum likelihood phylogenetic tree was constructed using IQ-TREE with the parameters iqtree -s a -alrt 1000 -bb 1000 -nt AUTO.

Reference sequences of RuBisCo large subunit were obtained from a previous study (26). All sequences were aligned using MAFFT v6.864b (91). Poorly aligned regions were removed using TrimAl v1.4.rev22 (86). The unrooted phylogeny was generated using RAxML v7.2.7 (86) with the following parameters: -f a -n boot -m PROTGAMMAIJTT -c 4 -e 0.001 -p 13452 -x 1165 -# 1000.

Reference sequences of hydrogenases were selected from a previous study (28). Alignments were generated using MUSCLE v3.8.31 (85), and divergent regions were filtered using TrimAl (86). The same model and detailed parameters of RAxML as RuBisCo large subunit phylogeny were used to construct the phylogeny of hydrogenase sequences.

### Recruiting 16S rRNA from NCBI.

Seventeen 16S rRNA gene sequences recovered from currently available “*Ca.* Aenigmarchaeota” genomes were used as input to search against the NCBI-nt (https://www.ncbi.nlm.nih.gov/) database via the BLASTn program with the default parameters. BLAST hits with coverage of >95% and identity of >85% were kept for the phylogenetic tree construction. Sequences were aligned, while poor alignment regions were eliminated. A maximum likelihood-based phylogeny was generated using RAxML v7.2.7 (86). Finally, we identified 236 16S rRNA gene sequences of “*Ca.* Aenigmarchaeota” by the result of phylogenetic analysis.

### Detection of horizontally transferred genes.

Twenty genomes with completeness of >75% and contamination of <5% evaluated by CheckM were taken into consideration for the inferences of horizontal gene transfers (HGTs). As a result, three SAGs were removed because of the low quality. Putative HGTs were identified for each genome using HGTector v2 (92). To determine the interaction partners or possible hosts of “*Ca.* Aenigmarchaeota,” recent horizontal gene transfers were identified by applying BLAST searches against the genome bins reconstructed from the corresponding communities. Only genes from outside DPANN are considered the so-called interphylum HGTs. Due to a lack of representative archaeal genomes in the prebuilt default database, especially the genomes from DPANN and TACK superphyla, 3,358 genomes were downloaded from the RefSeq database on 14 May 2019. Genome quality was evaluated using CheckM except microbes from the DPANN superphylum. Genome quality of the DPANN superphylum was performed using the same procedure as mentioned above. Genomes with completeness of <80% and contamination of >10% were discarded. The remaining high-quality genomes were dereplicated at the phylum level using dRep v2.3.2 (93). Finally, 1,133 genomes were picked out, and 689 of those genomes complementary to the default database were appended. The combined sequences were compiled into a database using DIAMOND (77), and the relevant taxonomy files were changed correspondingly. Then, the “search” step was performed for the 20 high-quality “*Ca.* Aenigmarchaeota” genomes with default parameters. During the “analyze” step, genomes belonging to the DPANN superphylum (taxonomy ID, 1783276) were treated as “closeTax,” but different species were used as “selfTax.” Specifically, *Aenigmarchaeum subterraneum* SCGC AAA011-O16 (taxonomy ID, 743730) was set as the “selfTax” for all MAGs for DRTY-6_2 bin 215 and JZ-2_2 bin 245; *Aenigmarchaeota archaeon* ex4484_224 (taxonomy ID, 2012503) was used as “selfTax” for GMQ_1 bin 18-1 and QQ_2 bin 128; *Aenigmarchaeota archaeon* ex4484_14 (taxonomy ID, 2012502) was used as “selfTax” for DRTY-6_2 bin 201; and *Aenigmarchaeota archaeon* JGI 0000106-F11 (taxonomy ID, 1130284) was used for DRTY-6_1 bin 65, DRTY-7_1 bin 34, and DRTY-6_2 bin 202. The identified interphylum HGTs for each genome were visualized using SankeyMATIC (http://sankeymatic.com/).

In more detail, preliminary genome binning was conducted as described above for each “*Ca.* Aenigmarchaeota”-containing microbial community. The taxonomic information was determined using GTDB-tk v.0.2.2 (94). Then, gene calling was performed for all genome bins. The predicted putative coding sequences were formatted into a BLAST database. A BLAST search against this database was conducted with genes identified from “*Ca.* Aenigmarchaeota” as input. Only BLAST hits from outside DPANN with a sequence identity of ≥70% and aligned region of ≥100 amino acids were retained. The target genes and BLAST hits that represented the first or last genes of the belonging scaffolds (≥5,000 bp) were discarded. The remaining genes were used for inferring the potential functional interactions between putative hosts and symbionts.

### Phylogenetic analysis of four horizontally transferred genes. (i) Reverse gyrase (*rgy*).

Scaffolds identified as *rgy* in “*Ca.* Aenigmarchaeota” were collected and used as input to blast against the NCBI-nr database (E value of <1E−5). The top 50 hits for each query were kept. The obtained *rgy* genes for all queries were combined and clustered using CD-HIT v4.6 (95), with a sequence identity cutoff of 90%. The identified representative sequences were used to build the phylogenetic tree. Sequences were aligned using MUSCLE, and poorly aligned regions were eliminated using TrimAl. Phylogeny was generated using IQ-TREE v1.6.11 (87) by integrating 1,000 times with the best model “LG+F+R9.” The generated phylogenetic tree was rooted using MAD v2.2 (96) with the default parameters.

*Superoxide dismutase (SOD2)*. The same procedures as the *rgy* phylogeny construction were applied, including sequence recruitment, clustering, alignment, and poorly aligned region elimination, except the sequence identity cutoff was set to 0.8 during the clustering using CD-HIT. The phylogenetic tree was generated using IQ-TREE with the best model “WAG+R10.”

### (ii) 8-oxo-dGTP diphosphatase (*mutT*).

The same procedures as the *rgy* phylogeny construction were applied, including sequence recruitment, clustering, alignment, and poorly aligned region elimination, except the sequence identity cutoff was set to 0.65 during the clustering using CD-HIT. The phylogenetic tree was generated using IQ-TREE with the best model “VT+F+R10.”

### (iii) Transcription initiation factor IIB (*TFIIB*).

The same procedures as the *rgy* phylogeny construction were applied, including sequence recruitment, clustering, alignment, and poorly aligned region elimination. The phylogenetic tree was generated using IQ-TREE with the best model “LG+R10.”

### Network-based co-occurrence analysis.

A total of 88 hot spring samples across the time and spatial scale were used to reveal the co-occurrence pattern of “*Ca.* Aenigmarchaeota” with other community members (Data Set S4). Metagenomic sequencing was conducted for all samples. Detailed quality control and assembly steps were done as described above. Genome binning was conducted for each community (as described above), and only genome bins with completeness of >50%, contamination of <5%, and *rpS3* gene called by AMPHORA2 (84) occurring exactly once in one genome were taken into consideration. The corresponding nucleotide sequences were extracted for the later analysis. Taxonomic information of each bin was obtained using GTDBtk v.0.2.2 (94). All predicted *rpS3* gene sequences from different data sets were combined and clustered into OTUs using USEARCH 9.2.84 with the following parameters: -cluster_smallmem -id 0.95. Taxonomy of the representative sequences was assigned according to the taxonomic information of belonging bins. The sequence depth of each *rpS3* gene sequence was used to build the OTU table and was calculated by mapping clean reads in each sample to the dereplicated *rpS3* gene sequences. Specifically, clean reads from each sample were mapped to *rpS3* gene sequences using BBmap with the same parameter settings as described above. The generated .bam files were sorted using SAMtools v.1.3.1 (97). Sequence depth was subsequently calculated using the “jgi_summarize_bam_contig_depths” program in MetaBAT. OTUs that occurred in less than six samples were filtered out to reduce the complexity, and 844 OTUs were kept for the subsequent network construction. The co-occurrence network was constructed using SparCC (98) with the default parameters, and 100 bootstrap samples were used to infer pseudo-*P* values. Those significant (*P < *0.05, two-sided) and robust correlations (rho > 0.6) between pairwise OTUs were used to infer a reliable network. Network visualization and relevant parameter calculations regarding modularity, betweenness, closeness, average clustering coefficient, average weighted degree, and average shortest path length were conducted in Gephi v.0.9.2 (99).

### Data availability.

All genomes in our study are available at Joint Genome Institute (JGI) IMG-MER under the study ID Gs0127627 and whole-genome sequencing accession numbers Ga0181641 (unclassified Aenigmarchaeota DRTY7 bin_34), Ga0181640 (unclassified Aenigmarchaeota DRTY6 bin_65), Ga0181639 (unclassified Aenigmarchaeota GMQ bin_18-1), Ga0227293 (unclassified Aenigmarchaeota JZ-2 bin_245), Ga0227294 (unclassified Aenigmarchaeota DRTY-6 bin_215), Ga0261588 (unclassified Aenigmarchaeota DRTY-6 bin_201), Ga0261590 (unclassified Aenigmarchaeota DRTY-6 bin_202), and Ga0261591 (unclassified Aenigmarchaeota QQ bin_128). All genomes are also available in the NCBI database. The BioProject number is PRJNA544494. The genome accession numbers are JAHLMM000000000 (Aenigmarchaeota_DRTY-6_1_bin_65), JAHLMN000000000 (Aenigmarchaeota_DRTY-7_1_bin_34), JAHLMO000000000 (Aenigmarchaeota_GMQ_1_bin_18-1), JAHLMP000000000 (Aenigmarchaeota_DRTY-6_2_bin_201), JAHLMQ000000000 (Aenigmarchaeota_DRTY-6_2_bin_202), JAHLMR000000000 (Aenigmarchaeota_DRTY-6_2_bin_215), JAHLMS000000000 (Aenigmarchaeota_JZ-2_2_bin_245), and JAHLMT000000000 (Aenigmarchaeota_QQ_2_bin_128).
