# Supplementary figures and images for: Deciphering Symbiotic Interactions of “Candidatus Aenigmarchaeota” with Inferred Horizontal Gene Transfers and Co-occurrence Networks
Source: mSystems. 2021 Jul 27;6(4):e00606-21. doi: 10.1128/mSystems.00606-21 (PMC8407114; doi:10.1128/mSystems.00606-21)

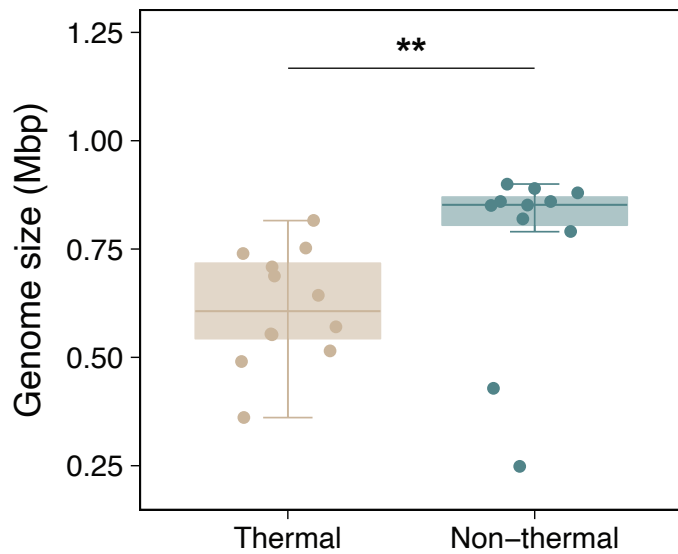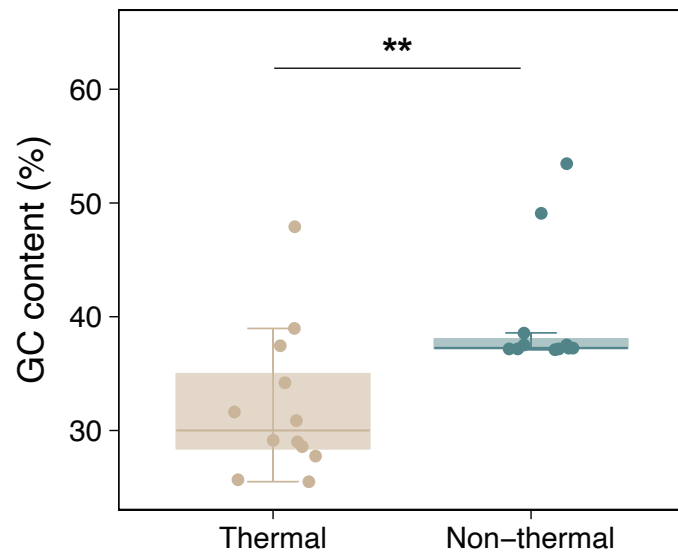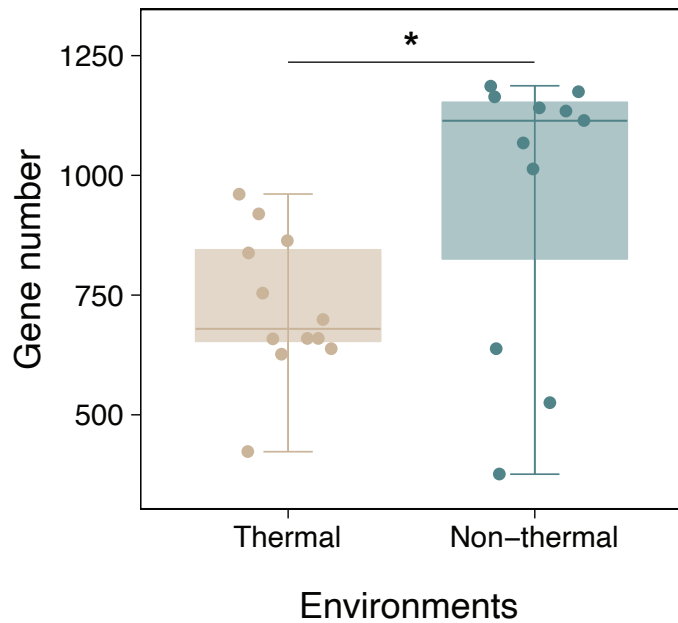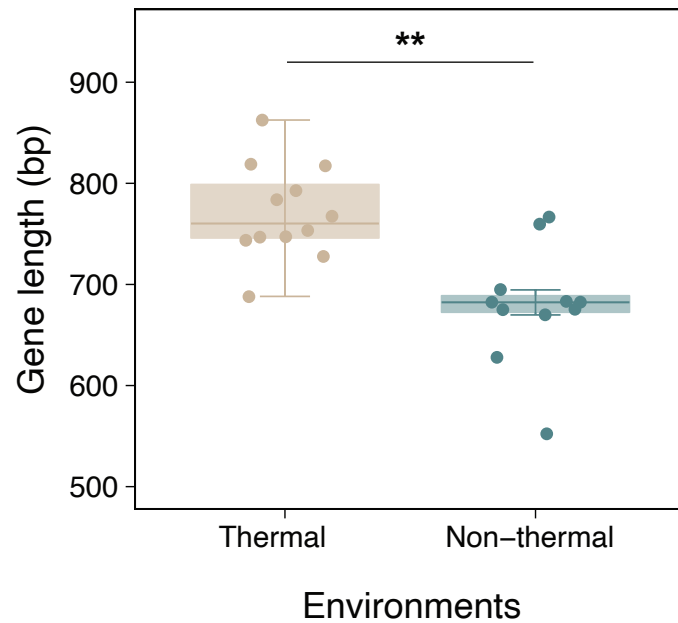

Supplement: FIG S1 [file msystems.00606-21-sf001.pdf]

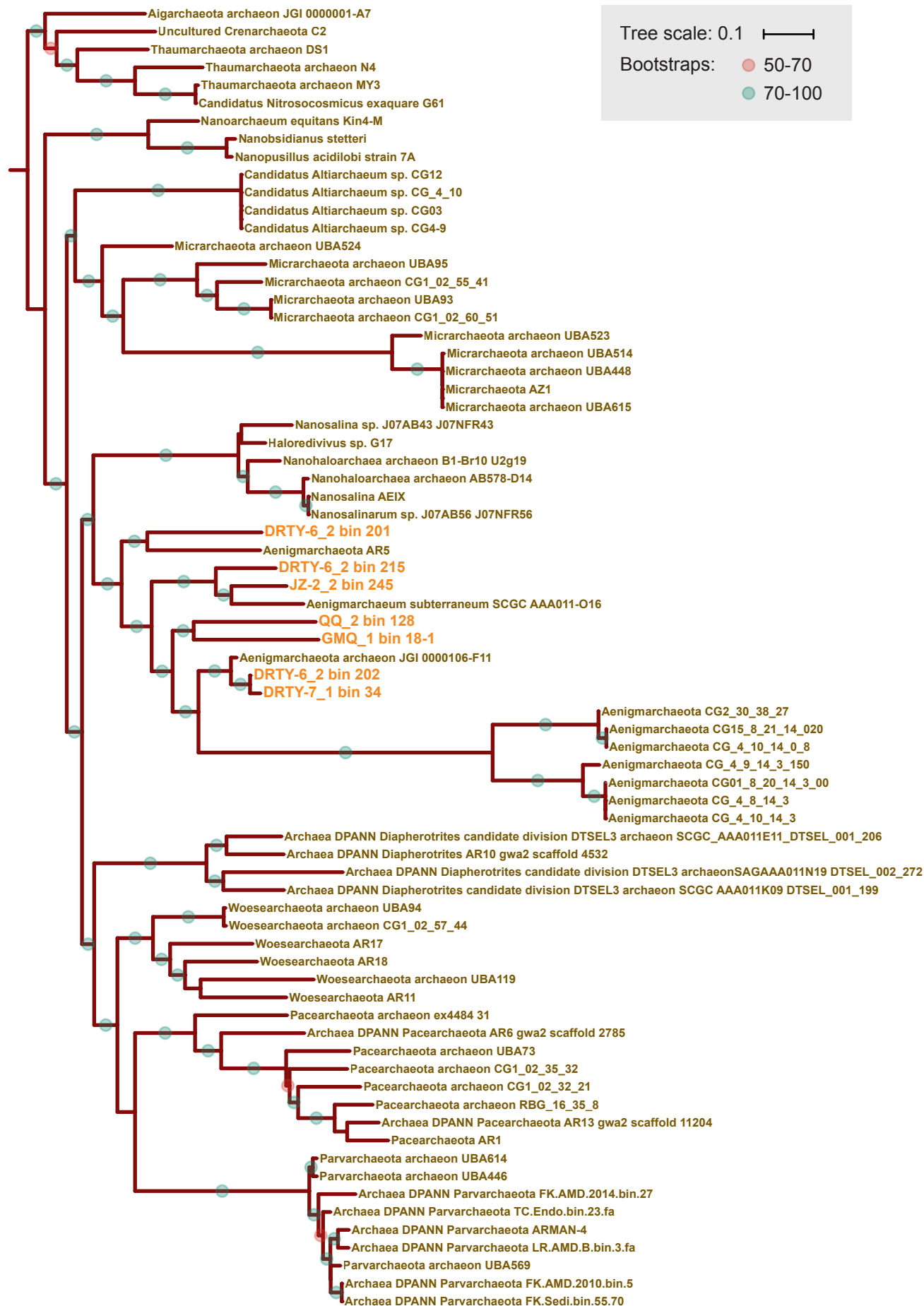

Supplement: FIG S2 [file msystems.00606-21-sf002.pdf]

a

## KOs

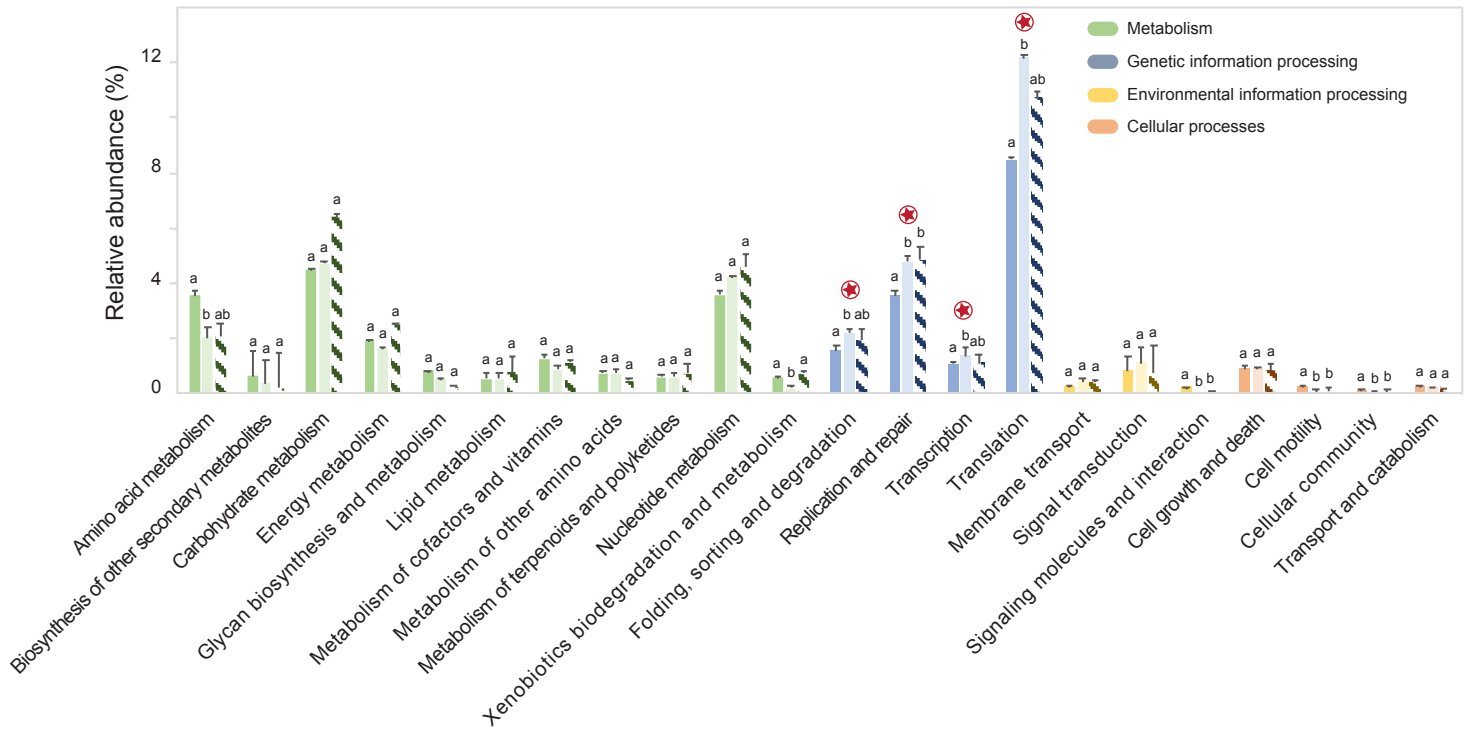

b

## arCOGs

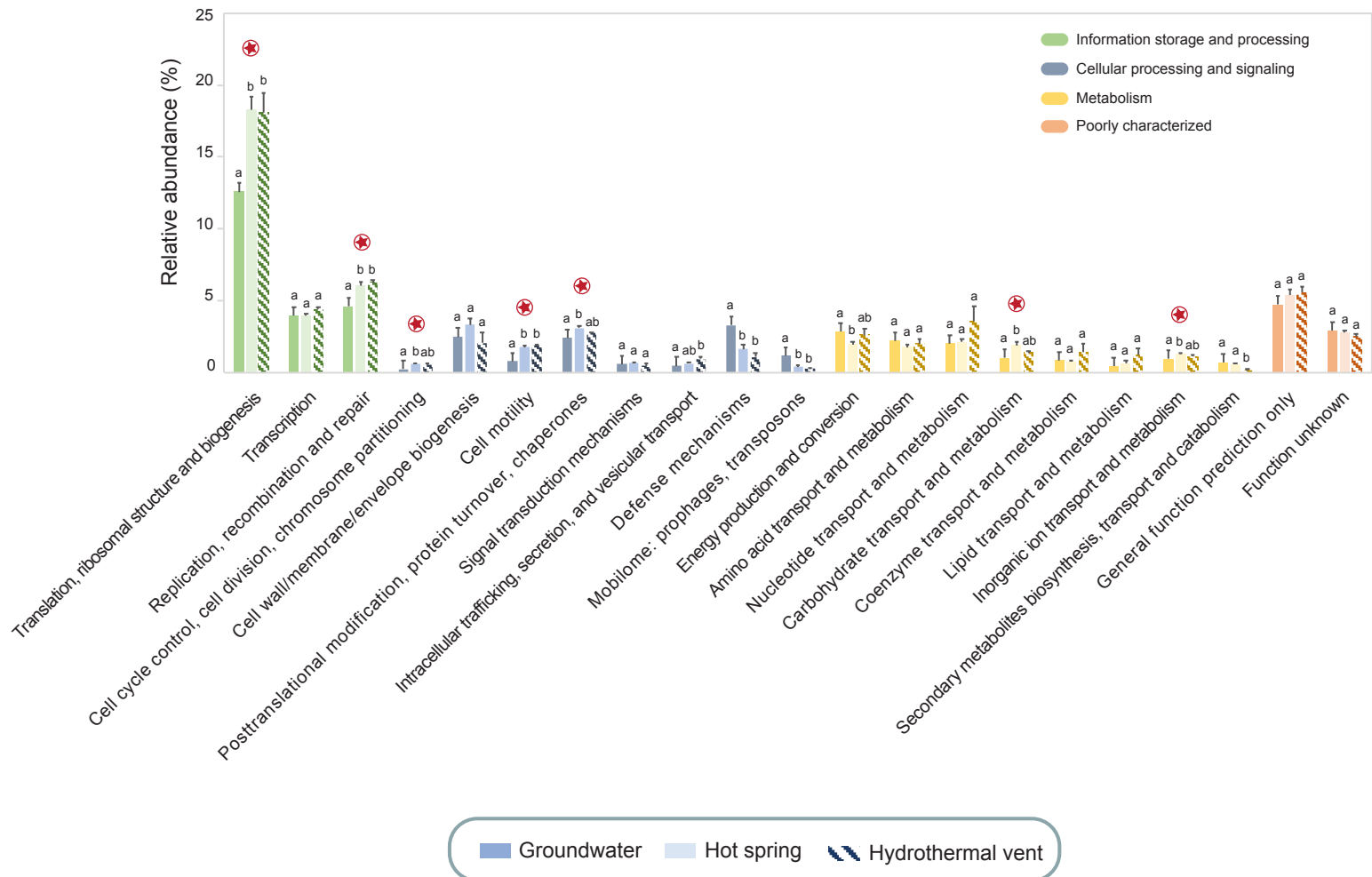

Supplement: FIG S3 [file msystems.00606-21-sf003.pdf]
