# Supplementary material for: Deciphering Symbiotic Interactions of “Candidatus Aenigmarchaeota” with Inferred Horizontal Gene Transfers and Co-occurrence Networks
Source: mSystems. 2021 Jul 27;6(4):e00606-21. doi: 10.1128/mSystems.00606-21 (PMC8407114; doi:10.1128/mSystems.00606-21)

a. CRISPR-cas system

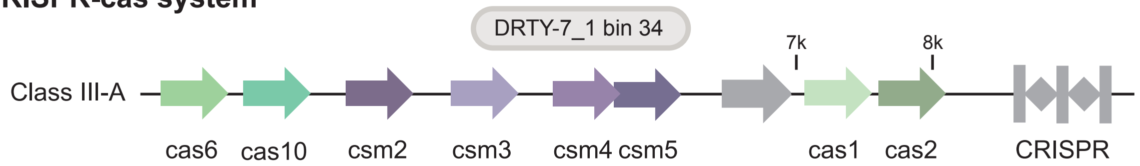

b. Restriction modification system

MAGs	Type I	Type II	Type III
DRTY-6_1 bin 65		★	★
DRTY-7_1 bin 34		★	★
GMQ_1 bin 18-1			★
QQ_2 bin 128		★	★
DRTY-6_2 bin 201	★	★	
DRTY-6_2 bin 202	★	★	★
Aenigmarchaeum subterraneum SCGC AAA011-O16	★		
Aenigmarchaeota AR5		★	
Aenigmarchaeota CG_4_10_14_0_8			★
Aenigmarchaeota CG_4_10_14_3			★
Aenigmarchaeota CG_4_8_14_3			★
Aenigmarchaeota CG_4_9_14_3_150			★
Aenigmarchaeota CG01_8_20_14_3_00			★
Aenigmarchaeota CG15_8_21_14_020	★		★
Aenigmarchaeota archaeon CG1_02_38_14			★
Aenigmarchaeota archaeon ex4484_56			★
Aenigmarchaeota archaeon ex4484_224			★
Aenigmarchaeota archaeon ex4484_14			★

c. Other potential cell defense systems

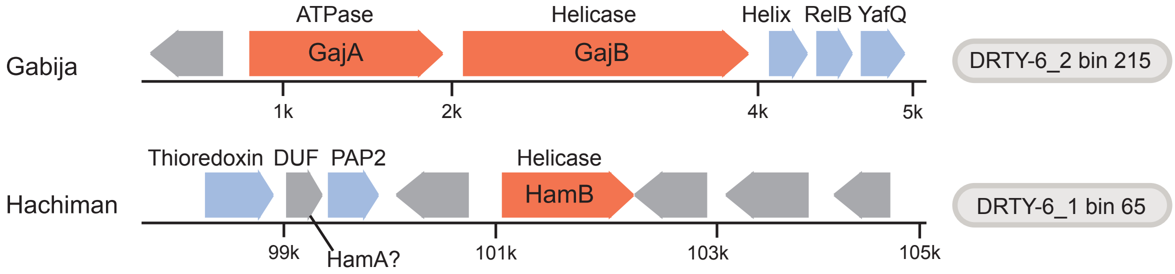

Supplement: FIG S4 [file msystems.00606-21-sf004.pdf]
