# Supplementary material for: Deciphering Symbiotic Interactions of “Candidatus Aenigmarchaeota” with Inferred Horizontal Gene Transfers and Co-occurrence Networks
Source: mSystems. 2021 Jul 27;6(4):e00606-21. doi: 10.1128/mSystems.00606-21 (PMC8407114; doi:10.1128/mSystems.00606-21)
Supplement: FIG S5 [file msystems.00606-21-sf005.pdf]

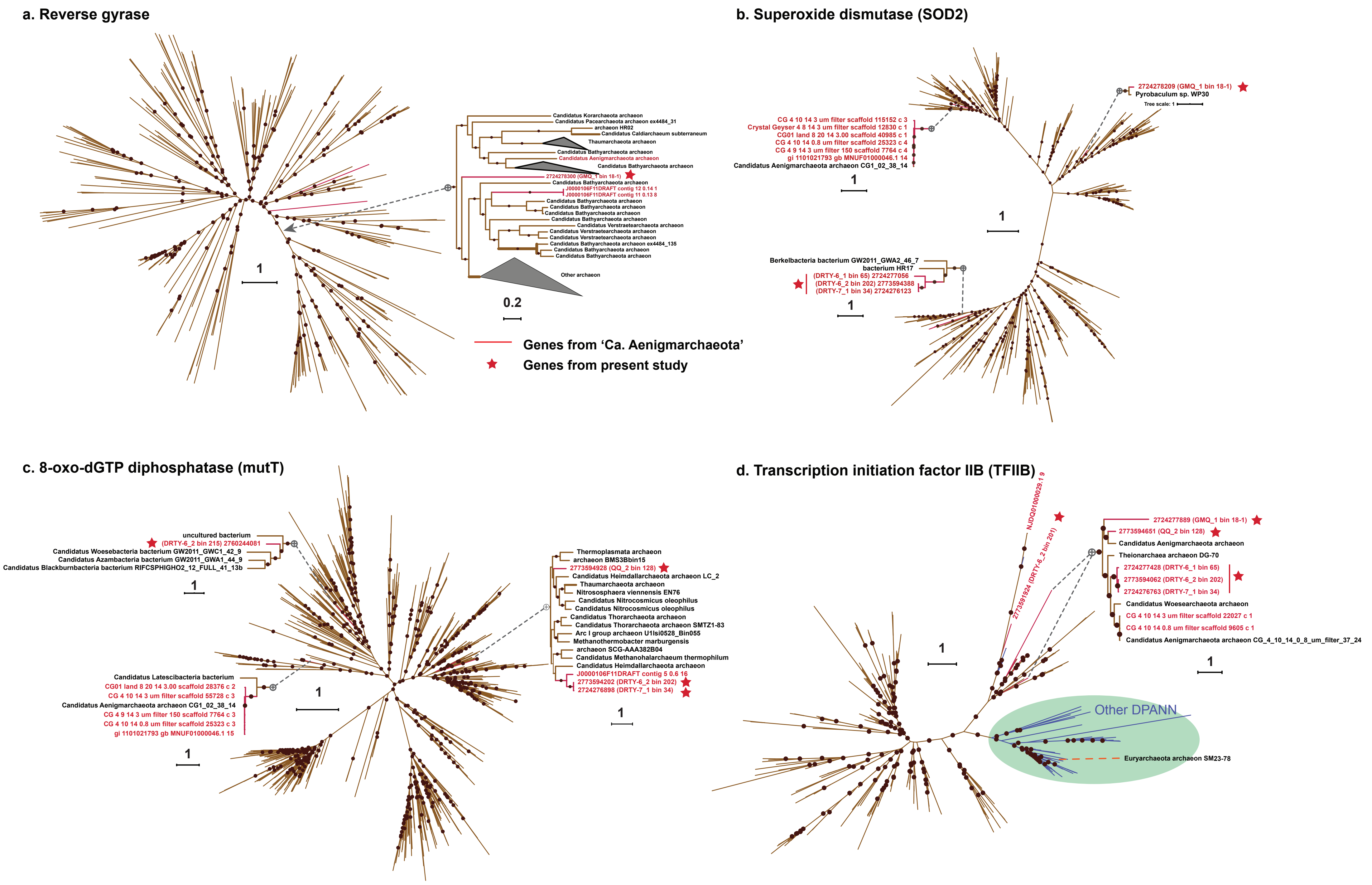
a. Reverse gyrase
1
Candidatus Korarchaeota archaeon
Candidatus Pacearchaeota archaeon ex4484_31
archaeon HR02
Candidatus Caldiarchaeum subterraneum
Thaumarchaeota archaeon
Candidatus Bathyarchaeota archaeon
Candidatus Aenigmarchaeota archaeon
Candidatus Bathyarchaeota archaeon
2724278300 (GMQ_1 bin 18-1)
Candidatus Bathyarchaeota archaeon
J0000106F11DRAFT contig 12 0.14 1
J0000106F11DRAFT contig 11 0.13 8
Candidatus Bathyarchaeota archaeon
Candidatus Bathyarchaeota archaeon
Candidatus Bathyarchaeota archaeon
Candidatus Bathyarchaeota archaeon
Candidatus Verstraetearchaeota archaeon
Candidatus Verstraetearchaeota archaeon
Candidatus Verstraetearchaeota archaeon
Candidatus Bathyarchaeota archaeon ex4484_135
Candidatus Bathyarchaeota archaeon
Candidatus Bathyarchaeota archaeon
Other archaeon
0.2
Genes from ‘Ca. Aenigmarchaeota’
Genes from present study
b. Superoxide dismutase (SOD2)
CG 4 10 14 3 um filter scaffold 115152 c 3
Crystal Geyser 4 8 14 3 um filter scaffold 12830 c 1
CG01 land 8 20 14 3.00 scaffold 40985 c 1
CG 4 10 14 0.8 um filter scaffold 25323 c 4
CG 4 9 14 3 um filter 150 scaffold 7764 c 4
gi 1101021793 gb MNUF01000046.1 14
Candidatus Aenigmarchaeota archaeon CG1_02_38_14
1
2724278209 (GMQ_1 bin 18-1)
Pyrobaculum sp. WP30
Tree scale: 1
1
Berkelbacteria bacterium GW2011_GWA2_46_7
bacterium HR17
(DRTY-6_1 bin 65) 2724277056
(DRTY-6_2 bin 202) 2773594388
(DRTY-7_1 bin 34) 2724276123
1
c. 8-oxo-dGTP diphosphatase (mutT)
uncultured bacterium
(DRTY-6_2 bin 215) 2760244081
Candidatus Woesebacteria bacterium GW2011_GWC1_42_9
Candidatus Azambacteria bacterium GW2011_GWA1_44_9
Candidatus Blackburnbacteria bacterium RIFCSPHIGHO2_12_FULL_41_13b
1
Candidatus Latescibacteria bacterium
CG01 land 8 20 14 3.00 scaffold 28376 c 2
CG 4 10 14 3 um filter scaffold 55728 c 3
Candidatus Aenigmarchaeota archaeon CG1_02_38_14
CG 4 9 14 3 um filter 150 scaffold 7764 c 3
CG 4 10 14 0.8 um filter scaffold 25323 c 3
gi 1101021793 gb MNUF01000046.1 15
1
1
Thermoplasmata archaeon
archaeon BMS3Bbin15
2773594928 (QQ_2 bin 128)
Candidatus Heimdallarchaeota archaeon LC_2
Thaumarchaeota archaeon
Nitrososphaera viennensis EN76
Candidatus Nitrocosmicus oleophilus
Candidatus Nitrocosmicus oleophilus
Candidatus Thorarchaeota archaeon
Candidatus Thorarchaeota archaeon SMTZ1-83
Arc I group archaeon U1lsi0528_Bin055
Methanothermobacter marburgensis
archaeon SCG-AAA382B04
Candidatus Methanohalarchaeum thermophilum
Candidatus Heimdallarchaeota archaeon
J0000106F11DRAFT contig 5 0.6 16
2773594202 (DRTY-6_2 bin 202)
2724276898 (DRTY-7_1 bin 34)
1
d. Transcription initiation factor IIB (TFIIB)
NJDQ01000029.1 9
2773591924 (DRTY-6_2 bin 201)
1
2724277889 (GMQ_1 bin 18-1)
2773594651 (QQ_2 bin 128)
Candidatus Aenigmarchaeota archaeon
Theionarchaea archaeon DG-70
2724277428 (DRTY-6_1 bin 65)
2773594062 (DRTY-6_2 bin 202)
2724276763 (DRTY-7_1 bin 34)
Candidatus Woesearchaeota archaeon
CG 4 10 14 3 um filter scaffold 22027 c 1
CG 4 10 14 0.8 um filter scaffold 9605 c 1
Candidatus Aenigmarchaeota archaeon CG_4_10_14_0_8_um_filter_37_24
1
Other DPANN
Euryarchaeota archaeon SM23-78
